# Detecting multiple differentially methylated CpG sites and regions related to dimensional psychopathology in youths

**DOI:** 10.1186/s13148-019-0740-z

**Published:** 2019-10-21

**Authors:** Leticia M. Spindola, Marcos L. Santoro, Pedro M. Pan, Vanessa K. Ota, Gabriela Xavier, Carolina M. Carvalho, Fernanda Talarico, Patrick Sleiman, Michael March, Renata Pellegrino, Elisa Brietzke, Rodrigo Grassi-Oliveira, Jair J. Mari, Ary Gadelha, Euripedes C. Miguel, Luis A. Rohde, Rodrigo A. Bressan, Diego R. Mazzotti, João R. Sato, Giovanni A. Salum, Hakon Hakonarson, Sintia I. Belangero

**Affiliations:** 1 Genetics Division, Department of Morphology and Genetics, Universidade Federal de São Paulo (UNIFESP), Rua Botucatu 740, Ed. Leitão da Cunha, Vila Clementino, Sao Paulo, SP Brazil; 2 LiNC - Laboratory of Integrative Neuroscience, UNIFESP, São Paulo, Brazil; 3 Department of Psychiatry, UNIFESP, São Paulo, Brazil; 4 Center for Applied Genomics, The Children’s Hospital of Philadelphia, Philadelphia, USA; 5 Brain Institute, Pontifícia Universidade Católica do Rio Grande do Sul (PUCRS), Porto Alegre, Brazil; 6 Department of Psychiatry, Faculdade de Medicina da Universidade de São Paulo (FMUSP), São Paulo, Brazil; 7 Department of Psychiatry, Hospital de Clínicas de Porto Alegre, Universidade Federal do Rio Grande do Sul (UFRGS), Porto Alegre, Brazil; 8 Center for Sleep and Circadian Neurobiology, University of Pennsylvania, Philadelphia, USA; 9 Center of Mathematics, Computing and Cognition, Universidade Federal do ABC, Santo André, Brazil

**Keywords:** Mental disorders, Epigenetics, Methylation, Gene expression, Transcription

## Abstract

**Background:**

Psychiatric symptomatology during late childhood and early adolescence tends to persist later in life. In the present longitudinal study, we aimed to identify changes in genome-wide DNA methylation patterns that were associated with the emergence of psychopathology in youths from the Brazilian High-Risk Cohort (HRC) for psychiatric disorders. Moreover, for the differentially methylated genes, we verified whether differences in DNA methylation corresponded to differences in mRNA transcript levels by analyzing the gene expression levels in the blood and by correlating the variation of DNA methylation values with the variation of mRNA levels of the same individuals. Finally, we examined whether the variations in DNA methylation and mRNA levels were correlated with psychopathology measurements over time.

**Methods:**

We selected 24 youths from the HRC who presented with an increase in dimensional psychopathology at a 3-year follow-up as measured by the Child Behavior Checklist (CBCL). The DNA methylation and gene expression data were compared in peripheral blood samples (*n* = 48) obtained from the 24 youths before and after developing psychopathology. We implemented a methodological framework to reduce the effect of chronological age on DNA methylation using an independent population of 140 youths and the effect of puberty using data from the literature.

**Results:**

We identified 663 differentially methylated positions (DMPs) and 90 differentially methylated regions (DMRs) associated with the emergence of psychopathology. We observed that 15 DMPs were mapped to genes that were differentially expressed in the blood; among these, we found a correlation between the DNA methylation and mRNA levels of *RB1CC1* and a correlation between the CBCL and mRNA levels of *KMT2E.* Of the DMRs, three genes were differentially expressed: *ASCL2*, which is involved in neurogenesis; *HLA-E*, which is mapped to the MHC loci; and *RPS6KB1*, the gene expression of which was correlated with an increase in the CBCL between the time points.

**Conclusions:**

We observed that changes in DNA methylation and, consequently, in gene expression in the peripheral blood occurred concurrently with the emergence of dimensional psychopathology in youths. Therefore, epigenomic modulations might be involved in the regulation of an individual’s development of psychopathology.

## Background

Mental disorders contribute significantly to the global burden of disease, ranking among the top 10 causes of disability in developed countries worldwide [1]. Recent research approaches have focused on describing the genetic contribution to these disorders. The advent of large-scale genome-wide association studies (GWAS) has enabled the hypothesis-free exploration of genetic risk factors. These endeavors have been highly successful: a recent large-scale GWAS meta-analysis identified 102 independent genomic loci exhibiting a genome-wide significant association with depression, a psychiatric disorder with relatively low heritability [2]. Moreover, a recent study that quantified the genetic sharing of 25 brain disorders from GWAS identified a significant sharing of common variant risk among mental disorders and between mental disorders and brain phenotypes, including cognitive measures [3].

However, there is considerable uncertainty regarding whether genes identified in GWAS are causally involved in the pathogenesis of mental disorders and how their functions are regulated. Since many factors could impact gene expression, the field of epigenomic variation, including DNA methylation, has evolved into a sophisticated perspective on the basic mechanisms of gene regulation, which occur at the interface between a static genome and a dynamic environment [4]. DNA methylation is the best-characterized epigenetic modification, influencing gene expression via the disruption of transcription factor binding in promoter regions and the recruitment of methyl-binding proteins that initiate chromatin compaction and gene silencing [5]. Methylation primarily occurs at cytosine residues within CpG dinucleotides, and it represents a true epigenetic mechanism since it is faithfully maintained in the absence of the inducing signal, i.e., it is heritable.

There is a vast literature on DNA methylation studies in mental disorders [6, 7]. However, the dynamic nature of epigenetic modification means that a range of potentially important confounding factors needs to be considered, including tissue or cell type, age, sex, and lifestyle exposures [8, 9]. Moreover, most studies to date have two important limitations: cross-sectional designs, limiting the inferences that can be drawn about the etiological processes; and the use of adult samples with psychiatric disorders, a clinical group with higher exposure to the disease process, medication, and environmental lifestyle confounders. In addition, the greatest burden of mental disorders occurs during childhood and adolescence [10], which are critical periods for brain development, plasticity, and maturation. Recent research implicates neurodevelopmental processes in the pathophysiology of several mental disorders, even in clinical syndromes that typically show their first symptoms in late stages of life, such as Alzheimer’s disease [11, 12]. Therefore, a longitudinal study of DNA methylation changes associated with psychopathology in a cohort of youths has considerable potential to be informative regarding the early mechanisms of mental disorders.

**Fig. 1:**
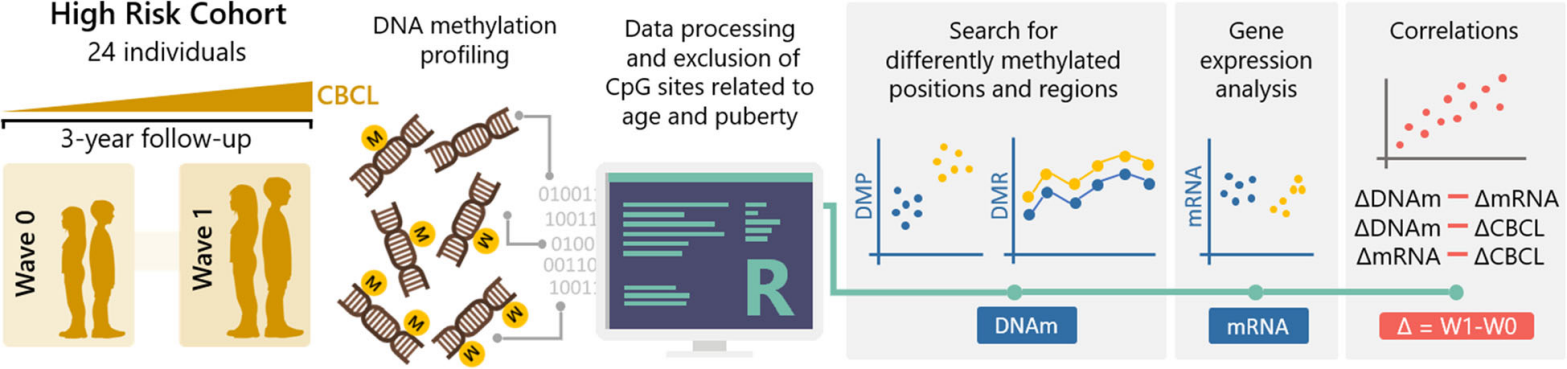
Overview of the study design. We selected 24 youths from the Brazilian High-Risk Cohort (HRC) for psychiatric disorders who presented with an increase in dimensional psychopathology after 3 years of follow-up as measured by the Child Behavior Checklist (CBCL). After implementing a methodological framework to reduce the effect of important confounders (chronological age and puberty) on the results, genome-wide DNA methylation was investigated in the context of both differentially methylated positions (DMPs) and differentially methylated regions (DMRs). Moreover, for differentially methylated genes, we verified whether differences in DNA methylation corresponded to differences in mRNA transcripts by analyzing gene expression levels in the blood from the same individuals. Finally, we examined whether there were correlations between (a) the variation in DNA methylation (ΔDNAm) and the variation in gene expression (ΔmRNA); (b) the variation in the total score of the CBCL (ΔCBCL) and ΔDNAm; and (c) ΔCBCL and ΔmRNA. The variations were calculated by subtracting wave 1 (W1) values from wave 0 (W0) values

In this longitudinal study with repeated blood sampling, we aimed to identify changes in the genome-wide DNA methylation patterns that are associated with the emergence of dimensional psychopathology during the transition from childhood to adolescence. Moreover, we tested whether differences in DNA methylation corresponded to differences in mRNA transcripts by analyzing the gene expression levels in the blood and by correlating the variation of DNA methylation values with the variation of mRNA levels in the same individuals. Finally, we examined whether variations in DNA methylation and mRNA levels were correlated with psychopathology measures across time (see Fig. 1).

## Methods

### Study procedures and participant selection

We selected a subsample from a large, prospective, community school-based study, the Brazilian High-Risk Cohort (HRC) for psychiatric disorders. The cohort characteristics and study design are detailed elsewhere [13] and in the Additional file 1. Briefly, we assessed subjects from two Brazilian cities (São Paulo and Porto Alegre) in two different waves: wave 0 (W0, representing the baseline) and wave 1 (W1, representing a 3-year follow-up). For both waves, the evaluations were performed over multiple visits, including a household parent interview and, on a separate visit, a collection of blood samples to assess peripheral biomarkers. In the household parent interview, the participants were assessed using a structured diagnostic interview, the Development and Well-Being Assessment (DAWBA) [14], to evaluate their psychiatric diagnosis according to the DSM-IV. On the day of the blood collection, dimensional psychopathology measures were assessed using the Child Behavior Checklist (CBCL) [15].

The CBCL is a widely used inventory that provides parent-report information on a wide array of behavioral problems in youths. See the Additional file 1 for more detail about the CBCL and validation literature from the CBCL. The Research Ethics Committee approved the research protocol. All parents and children/youths provided written informed consent before inclusion in the cohort. See the Additional file 1 for more detail about the CBCL.

From the pool of subjects with good-quality blood samples available for both waves, we selected subjects who met the following four criteria: (1) they lived in São Paulo (to exclude site effects) at both time-points; (2) they did not fulfill DSM-IV criteria for any mental disorder in the DAWBA and presented with low levels of dimensional psychopathology at the baseline (CBCL total score < 30.5 at W0); (3) they presented with high levels of dimensional psychopathology at the 3-year follow-up (CBCL total score ≥ 30.5 at W1); and (4) they presented with important changes in dimensional psychopathology levels between assessments (ΔCBCL = CBCL_W1_–CBCL_W0_ > 15). Cutoff values for the total CBCL were based on receiver operating characteristic (ROC) curve analysis using the CBCL score as a predictor of categorical mental disorders according to the DAWBA. The ROC analysis and cutoffs are described in Additional file 1. These criteria were met by 33 subjects, but only 24 had gene expression data available. Therefore, the final sample comprised 24 individuals from the HRC with low levels of dimensional psychopathology at W0, as measured by the CBCL total score, and high levels at W1.

### DNA methylation—Infinium MethylationEPIC BeadChip

A total of 10 mL of whole blood was collected in EDTA tubes (Becton Dickinson (BD), Franklin Lakes, NJ). Subsequently, DNA was isolated using a Gentra Puregene Kit (Qiagen) according to the manufacturer’s instructions. The DNA was treated with bisulfite, hybridized to Infinium MethylationEPIC BeadChips (“EPIC array”—Illumina, San Diego, CA), and scanned using the manufacturer’s protocol. To minimize systematic bias, the samples were randomly distributed onto the BeadChips, which held eight samples per BeadChip.

### Gene expression—HumanHT-12 v4 Expression BeadChips

Blood was collected using PAXgene RNA tubes (PreAnalytix, Hombrechtikon, Switzerland) and RNA was isolated using a PAXgene Blood RNA Kit (Qiagen, Stockach, Germany). A total of 200 ng of RNA was used with the Illumina® Total Prep™ RNA Amplification Kit (Life Technologies, Carlsbad, CA) to synthesize cRNA, which was hybridized to Human HT-12 v4 Expression BeadChips (Illumina).

### Quality control

**Fig. 2:**
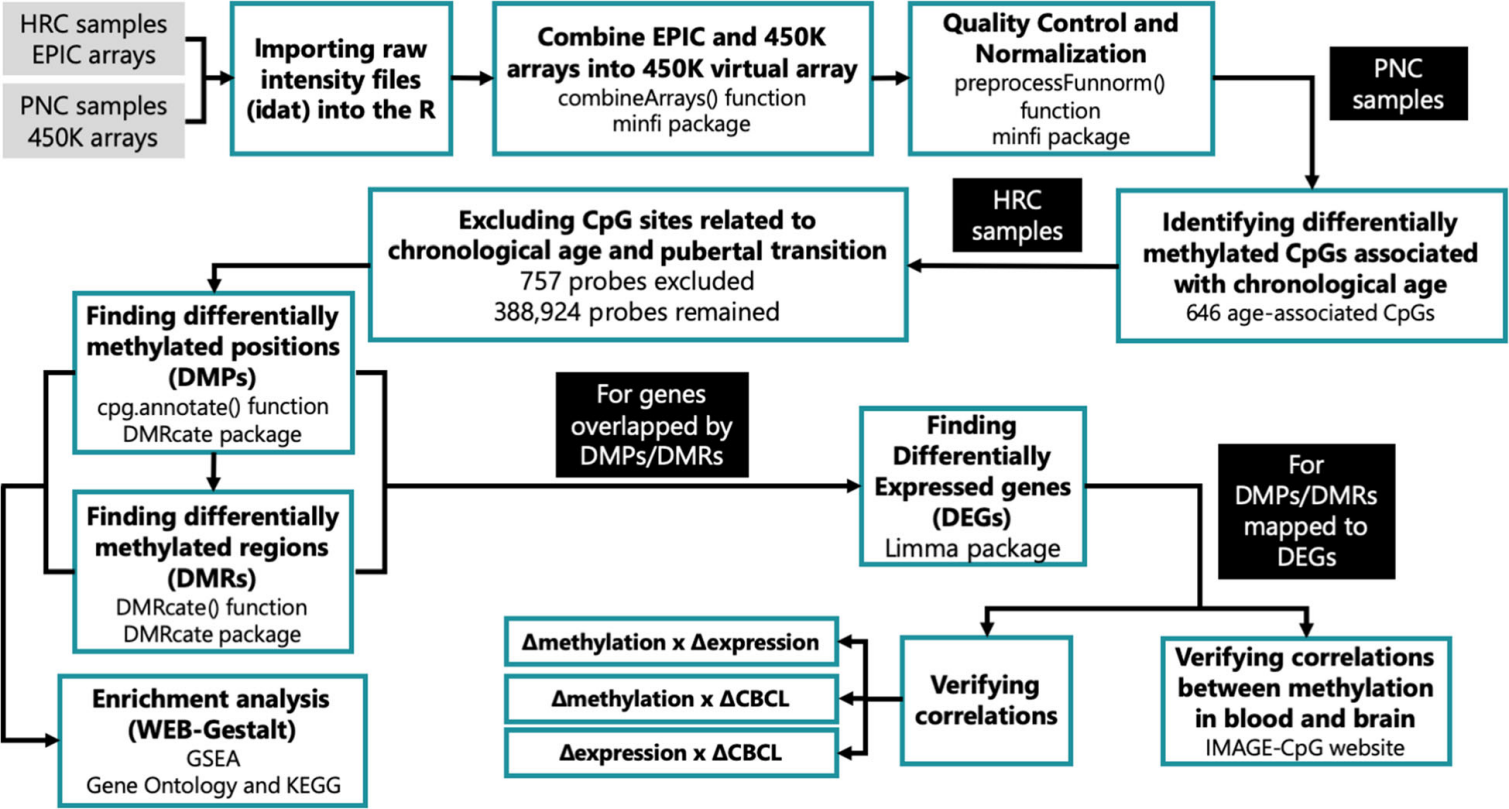
Flowchart of all the steps and analyses performed. HRC high-risk cohort. PNC Philadelphia Neurodevelopmental Cohort. DMPs differentially methylated positions. DMRs differentially methylated regions. DEGs differentially expressed genes. The other abbreviations are described in the text

All the steps and analyses performed are summarized in Fig. 2.

#### Genome-wide quantification of DNA methylation

The raw intensity files (idat) generated by the EPIC arrays were imported into the R programming environment (v3.5.1) using RStudio (v1.1.453). The methylation level at each CpG site was calculated as a beta value [β = methylated intensity/(methylated intensity + unmethylated intensity)], which varied from 0 (no methylation) to 1 (complete methylation). Before the quality control (QC), we converted the EPIC array (866,238 probes) into a 450 K virtual array (452,832 probes) in order to compare it to an independent population of youths and to the Almstrup et al. study [16].

The QC of the data was verified using different R packages and was adapted from Hannon et al. (2016) [17] and Maksimovic et al. (2016) [18]. We performed 12 QC steps, which are detailed in the Additional file 1. From these steps, four were to check: (i) the similarities and differences between the samples using multi-dimensional scaling (MDS) plots; (ii) the bisulfite conversion; (iii) the methylated and unmethylated signal intensities; and (iv) the samples/probes that failed based on detection *p* values.

Normalization of the DNA methylation data was performed using the preprocessFunnorm() function in the minfi package (v1.26.2) [19]. We did not find a relevant batch effect (Additional file 1: Figure S1). None of the HRC samples were excluded during QC, which totaled 48 biological samples from 24 individuals. From the 452,832 probes, 63,170 were excluded during QC, with 389,662 probes remaining for subsequent analysis.

#### Identification and exclusion of CpG sites related to chronological age and pubertal transition

Many studies have investigated the relationship between DNA methylation patterns and chronological age [20–22]. Aiming to reduce the influence of chronological age on our longitudinal results, we verified which DNA methylation markers were associated with age in an independent population of youths. Thus, we selected healthy youths from the Philadelphia Neurodevelopmental Cohort (PNC), which has been described elsewhere [23]. The selection criteria were as follows: (1) similar ancestry compared to the HRC, based on a principal component analysis (PCA) generated from the SNP array data (Additional file 1: Figure S2). For this analysis, we selected individuals who were within the ranges of − 0.035 < PC1 < 0.028 and PC2 > − 0.1. These ranges were chosen based on a visual inspection of the PC1 × PC2 plot; and (2) an age at the date of the blood collection within the age range of the HRC samples (from 7 to 17 years of age). Thus, the selection comprised 140 children and adolescents without psychiatric disorders. Raw intensity files generated by Infinium HumanMethylation450 BeadChips (450Karrays-Illumina) were imported into R, and QC was performed as for the HRC samples. The bisulfite conversion was not successful for two samples, and another sample showed lower methylated and unmethylated signal intensities (see Additional file 1). Therefore, these three samples were excluded, and we analyzed DNA methylation data from 137 individuals. Linear regression models were used to identify the probes associated with age (independent variable) without including any covariates. We excluded all CpG sites associated with chronological age from further analysis.

Based on Tanner’s classification [24], all 24 children from our sample had pubertal onset after W0 and before W1 blood collection. In a recent study, Almstrup et al. [16] reported that changes in single methylation sites in whole blood were associated with physiological pubertal transition and reproductive function. Therefore, we also excluded from the final analyses all CpGs associated with pubertal age (false discovery rate; FDR < 0.001), and all CpGs that were correlated with more than three of the five analyzed circulating reproductive hormones (FDR < 0.05) reported by Almstrup et al. (2016). Finally, as the PNC and Almstrup et al. assessed DNA methylation using 450 K arrays, we excluded all EPIC probes that were not included in the 450 K array.

#### Genome-wide quantification of gene expression

The raw data were pre-analyzed using GenomeStudio software and then imported into R. QC was performed using the lumi package (v2.32.0). We performed a background correction using the maximum likelihood estimation (MLE) [25] and, to ensure that the different BeadChips were comparable to one another, we used a robust spline normalization (RSN). The QC resulted in 6322 probes with high-quality data available for the differentially expressed gene analyses. After QC, two participants were excluded because the reported sex was different from predicted sex using probes on sexual chromosomes. Thus, 22 participants had available DNA methylation and gene expression data from both waves.

### Statistical analysis

#### DNA methylation

While β values were used for the visualization and interpretation of the results because the value range of methylation is easily interpretable, the *M* values [*M* value = log2(methylated intensity/unmethylated intensity)] were used for the differential methylation analysis [26]. To find DMPs, i.e., analysis for each CpG site, we used regression models with subjects included as dummy variables (comparison within-subjects). The outcome variables were the *M* values from each CpG, the independent variable was the time point (W0 or W1), and we adjusted for batch effects (BeadChip number and BeadChip position) to ensure that our analysis was not influenced by any kind of batch effect unidentified. To verify the influence of medication (use or not) on the results, we repeated the regression models using the batch effects and medication as covariates. To account for multiple testing, an FDR procedure using the Benjamini–Hochberg (BH) method [27] was applied, with values below 0.05 considered significant.

Although performing CpG-wise analysis is useful and informative, it is also important to know whether several proximal CpG sites are simultaneously differentially methylated, i.e., to identify the DMRs. We investigated the DMRs using the DMRcate package [28]. DMRcate identifies and ranks the most differentially methylated regions across the genome based on the tunable kernel smoothing method. A bandwidth of 1000 nucleotides (lambda = 1000) and a scaling factor of 2 (*C* = 2) were used as recommended by the DMRcate authors. The results were corrected for multiple comparisons using the BH method. Probe location and the gene annotation were taken from Illumina reference files. Annotation was performed according to hg19.

We checked whether the blood cell composition estimates were different between the waves using generalized estimating equations (GEE). Gaussian distribution and independence were used as the expected autocorrelation structure, and we found no significant differences between the waves. Moreover, we checked whether all participants had similar genetic ancestry by verifying whether the first two PCs generated from the SNP array data were correlated with the first 10 PCs from the methylation data, and did not find any significant correlation. As our sample size was small and the number of variables in the model could prevent us from identifying interesting findings purely owing to an overfitting problem, the cell-composition estimates and genetic ancestry were not added as covariates to the regression models. See the Additional file 1 for the full description.

#### Gene expression

Differential expression analyses were performed only for the genes that were mapped to or near to the DMPs/DMRs. We defined that a DMP/DMR was mapped near to a gene when a CpG site or region was located 0–1500 bases upstream of the transcriptional start site (TSS) of a gene. We used regression models with subjects included as dummy variables in which the expression levels were the outcome and the waves were the independent variable, adjusting for the RNA integrity number (RIN), cRNA input, and BeadChip number. We considered *p* values below 0.05 without correction for multiple comparisons to be significant.

#### DNA methylation, gene expression, and CBCL score correlation

To verify whether the methylation at the DMPs mapped to differential expressed genes was correlated with gene expression, a Pearson correlation between the ΔDMPs (*M* values DMP_W1_–M values DMP_W0_) and ΔmRNA levels (mRNA_W1_–mRNA_W0_) was used. For the DMRs mapped to differentially expressed genes, we first took the mean of the *M* values for all CpGs within a DMR (mDMR), and then used a Pearson correlation between the ΔmDMRs (mDMR_W1_–mDMR_W0_) and ΔmRNA levels.

To verify whether biological measures were correlated with psychopathology, a Pearson correlation was used for the ΔCBCL and the (i) ΔDMP, (ii) ΔmDMR, and (iii) ΔmRNA levels. We considered *p* values below 0.05 without correction for multiple comparisons as significant correlations.

### Correlation between methylation in blood and brain

To verify the correlation between DNA methylation in the whole blood and brain for the DMPs and DMRs, we used the IMAGE-CpG tool. This tool is based on the DNA methylation data for blood, saliva, and buccal and live brain tissue using an EPIC array [29]. We only verified the correlation between the brain and blood for DMPs/DMRs mapped to genes that were differentially expressed in the blood. We considered a correlation between methylation in the brain and blood for Spearman rho > 0.20.

### Enrichment analysis

Enrichment analysis was performed for the DNA methylation results using the tools available in the WEB-based GEne SeT AnaLysis Toolkit (WEB-Gestalt) [30], selecting Gene Set Enrichment Analysis (GSEA) as the enrichment method. We performed two enrichment analyses: one using the DMP results and the other using the DMR results. For both enrichment analyses, we used all the genes for which the DMPs/DMRs were mapped to or near to the genes. We uploaded the gene ID and metric table (see Additional file 1) into WEB-Gestalt, selecting 1000 permutations and setting the minimum and maximum number of genes in the category as 5 and 500, respectively, and the mean between duplicate genes as the collapse method. We performed analyses for enriched GO (gene ontology) and KEGG (Kyoto Encyclopedia of Genes and Genomes) pathways. FDR < 0.05 using the BH method was considered significant.

## Results

The demographic characteristics of the participants are given in Table 1, Additional file 1: Table S1, and Additional file 1: Figure S3. The CBCL total score was higher in the females as compared to the males in wave 0. However, as we performed comparisons within-subjects, this difference did not influence our results. There was no difference in the ΔCBCL between the males and females across time [ΔCBCL mean (SD): males = 29.93 (10.31); females = 28.40 (8.24); *p* = 0.551]. On the day of the blood collection in W1, two participants reported using medications, but they were not able to report what those medications were.

**Table 1:** Demographic and clinical characteristics of the participants

Waves	Variables	Females (*n* = 10)	Males (*n* = 14)	*p* value
W0	Age (mean years, SD)	10.66 (1.76)	9.99 (1.66)	0.364
	CBCL (mean total score; SD)	22.90 (5.57)	15.86 (8.16)	0.020
W1	Age (mean years, SD)	14.40 (1.75)	13.86 (1.57)	0.443
	CBCL (mean total score; SD)	51.30 (11.56)	45.79 (10.05)	0.240

The *p* values were calculated using Student’s *t* test. *W0* wave 0 represented the baseline, *W1* wave 1 represented a 3-year follow-up, *SD* standard deviation, *CBCL* Child Behavior Checklist

### CpG sites related to chronological age and puberty transition

Our initial analysis to quantify the genome-wide patterns of DNA methylation used the EPIC array and included covariates for batch effects. The top-ranked DMPs were annotated to *MEIOB* (cg14976596), *ANO2* (cg23363039), *NTRK3* (cg20664238), *KCNAB3* (cg14918082), and *VPS35* (cg10271672) (Additional file 2: Table S2), which have been previously associated with the biological events of puberty, such as meiotic recombination and primary follicle transition, or with age-correlated DNA methylation [22, 31–34]. As described in the “Methods” section, we excluded the CpG sites associated with age and puberty to address this issue. In total, 738 probes associated with chronological age (Additional file 2: Table S3) or the puberty transition were excluded, providing us with 388,924 probes for subsequent analysis (after exclusion of the EPIC probes that were not present in 450 K arrays). We divided the following results into DMPs and DMRs.

### DMPs associated with the emergence of dimensional psychopathology

**Fig. 3:**
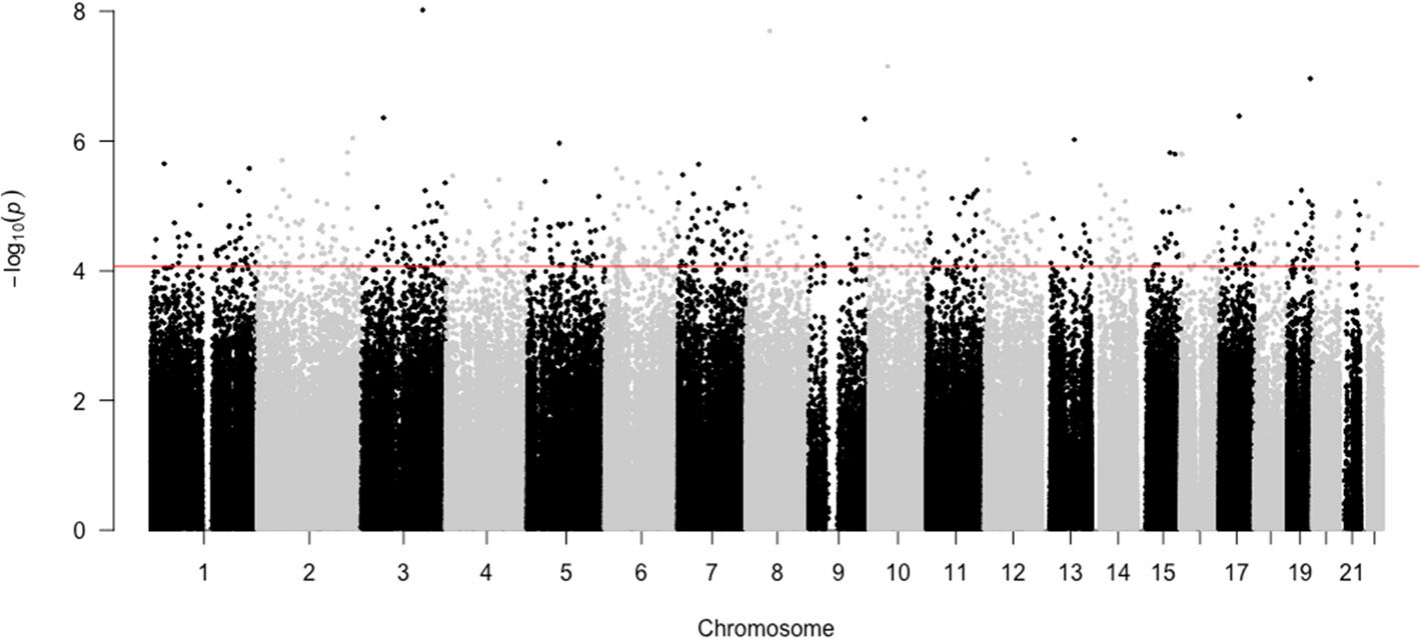
Manhattan plot showing the association *p* values (*y*-axis, −log10 scale) comparing the CpG methylation between W0 and W1 against the genomic location (*x*-axis) for the genome-wide analysis of the emergence of dimensional psychopathology in youths. Batch effects were used as covariates. Differentially methylated positions (DMPs) (above the red line, FDR < 0.05) are distributed across all autosomal chromosomes

After filtering the CpGs, we next repeated our genome-wide analysis using batch effects as covariates. We identified 663 DMPs associated with an increase in the CBCL total score. The DMPs were evenly distributed across all autosomes (Fig. 3 and Additional file 2: Table S4). The 20 top-ranked DMPs are given in Table 2. The 663 DMPs were annotated to or near to 531 genes, and these genes were used to search for significant enrichment. We found significant enrichment of the cell-cell adhesion via the plasma-membrane adhesion molecule GO biological process (GO: 0098742; FDR = 0.03) and of the post-synapse GO cellular component (GO: 0098794; FDR = 0.02) (Additional file 2: Table S5). To verify whether the use of the reported medications influenced our results, we repeated the analysis using batch effects and medication as covariates. The 663 DMPs associated with the emergence of dimensional psychopathology remained significant, indicating no influence of the reported medications on the results (Additional file 2: Table S6).

**Table 2:** The 20 most differentially methylated positions (DMPs) associated with the emergence of dimensional psychopathology

CpG ID	β fold change	Adjusted *p* value	Chromosome	Position (bp)	Gene annotation
cg11166600	0.1002	0.0037	chr3	143,695,141	*C3orf58* (body)
cg15427886	0.0896	0.0039	chr8	55,379,663	–
cg14116799	− 0.0764	0.0092	chr10	43,800,154	–
cg03213396	0.0699	0.0107	chr19	53,760,449	*VN1R2* (TSS1500)
cg25024195	0.0925	0.0253	chr17	46,144,968	–
cg03638479	− 0.0667	0.0253	chr3	52,009,768	*ABHD14B* (TSS1500); *ABHD14A* (body)
cg14525247	0.0630	0.0253	chr9	131,154,899	*MIR219–2* (body)
cg25275750	0.0562	0.0410	chr13	75,918,697	*TBC1D4* (body)
cg10544031	− 0.0261	0.0410	chr2	223,164,635	*PAX3* (TSS1500, 5′UTR); *CCDC140* (TSS1500)
cg22871870	0.0525	0.0415	chr15	77,348,431	*TSPAN3* (body)
cg20664238	0.0193	0.0415	chr15	88,798,877	*NTRK3* (body)
cg05673214	0.0498	0.0415	chr16	743,476	*FBXL16* (3′UTR)
cg01215511	0.0681	0.0414	chr16	2,975,552	*FLYWCH1* (5′UTR)
cg09288320	0.0770	0.0415	chr2	210,994,670	*C2orf67* (Body)
cg00137855	0.0612	0.0415	chr5	74,163,723	*FAM169A* (TSS1500)
cg19377326	0.0657	0.0416	chr1	31,486,063	*PUM1* (Body)
cg03729042	0.0444	0.0416	chr1	183,560,923	*NCF2* (TSS1500)
cg00589617	0.0647	0.0416	chr1	230,415,343	*GALNT2* (3’UTR)
cg24250902	0.0682	0.0416	chr1	230,415,547	*GALNT2* (3’UTR)
cg03754250	− 0.0394	0.0416	chr10	31,320,338	*ZNF438* (5’UTR, Body)

Wave 0 was used as the reference. Annotation was performed according to hg19, and the probe location and gene annotation were taken from Illumina reference files. *CpG ID* unique identifier from the Illumina CG database. *Adjusted p value*: false discovery rate (FDR) procedure using the Benjamini–Hochberg method (FDR < 0.05). *TSS1500* 200–1500 bases upstream of the transcriptional start site (TSS)

#### DMPs and gene expression

Of the 531 genes overlapped by DMPs, 103 had gene expression in the peripheral blood identified by a gene expression microarray, corresponding to 122 probe sets. We found that 15 genes (16 probe sets) were differentially expressed (Table 3 and Additional file 2: Table S7).

**Table 3:** Results of DNA methylation and gene expression for the genes in which CpG sites were associated with the emergence of dimensional psychopathology

*Gene*	DNA methylation				GENE EXPRESSION		
	CpG annotation	Gene annotation	FDR	β fold change	Probe ID	*p* value	Log fold change
*RASGRP1*	chr15:38,781,997	3′UTR	0.049	0.041	2,760,239	0.002	− 0.285
*BCL11A*	chr2:60,766,554	Body	0.043	0.069	6,580,450	0.005	−0.280
					3,170,440	0.014	−0.232
*ST13P4*	chr13:50,745,306	TSS1500	0.045	0.046	4,050,195	0.007	−0.236
*C7orf50*	chr7:1,053,726	Body	0.043	0.112	5,810,671	0.008	0.206
*WWP1*	chr8:87,427,271	Body	0.045	0.041	1,980,201	0.009	−0.204
*UBE3C*	chr7:156,951,457	Body	0.046	0.090	1,690,709	0.010	−0.156
*RB1CC1*	chr8:53,566,460	Body	0.045	0.086	3,890,092	0.012	−0.224
*PUM1*	chr1:31,486,063	Body	< 0.001	0.066	3,870,543	0.022	−0.162
*SMC3*	chr10:112,331,306	Body	0.047	0.059	2,970,292	0.024	−0.187
*RPS24*	chr10:79,793,501	TSS200	0.046	− 0.014	830,066	0.025	−0.593
*RPS6KB1*	chr17:57,970,021	TSS1500	0.045	− 0.027	1,940,576	0.026	−0.194
*KMT2E*	chr7:104,696,669	Body	0.045	0.050	3,120,050	0.033	0.249
*FAM217B*	chr20:58,520,201	3′UTR	0.044	0.024	770,221	0.039	0.228
*NIN*	chr14:51,295,867	5′UTR	0.043	0.078	5,310,717	0.043	−0.150
*HNRNPM*	chr19:8,513,923	Body	0.043	0.073	2,360,669	0.045	−0.150

Differentially methylated positions (DMPs) associated with the emergence of dimensional psychopathology in youths that had gene expression in peripheral venous blood identified by a *HumanHT-12 v4.0 Expression BeadChip* (Illumina). Probe location and gene annotation were taken from Illumina reference files. Annotation was performed according to hg19. Wave 0 (baseline) was used as the reference for both the DNA methylation and gene expression analyses. *Probe ID* unique identifier from the gene expression Illumina database. *TSS200* 0–200 bases upstream of the transcriptional start site (TSS). *TSS1500* 200–1500 bases upstream of the TSS. Official full names of the genes mentioned in the text: *BCL11A* B cell CLL/lymphoma 11A, *KMT2E* lysine methyltransferase 2E, *RB1CC1* RB1 inducible coiled-coil 1, *FAM217B* family with sequence similarity 217 member B

Regarding the DNA methylation correlation between the brain and blood for the DMPs mapped to these differentially expressed genes, previous analyses have shown that the methylation levels of 13 DMPs (86.7% of the total) are correlated to the levels in the brain (Additional file 2: Table S8) [29].

#### DMPs, gene expression, and CBCL score correlation

We verified whether the ΔmRNA levels of the 16 probe sets were correlated with ΔDMP methylation values (Additional file 2: Table S9). There was a correlation between the cg08517799 ΔDMP and *RB1CC1* ΔmRNA level (*r* = − 0.580; *p* = 0.005, Additional file 1: Figure S4). cg08517799 was mapped to the *RB1CC1* intron. Regarding the dimensional psychopathology, no ΔDMP values were correlated with the ΔCBCL scores (Additional file 2: Table S9). There was a correlation between the ΔCBCL and *KMT2E* ΔmRNA levels (*r* = 0.436; *p* = 0.042, Additional file 1: Figure S5), and also between the ΔCBCL and *FAM217B* ΔmRNA levels (*r* = 0.475; *p* = 0.025, Additional file 1: Figure S6).

### DMRs associated with the emergence of dimensional psychopathology

We searched for genomic regions (in a window of 1000 bases) where the DNA methylation was associated with the emergence of dimensional psychopathology in youths in a coordinated manner. We identified 90 DMRs that were annotated to or near to 86 genes and that spanned between 3 and 32 CpG sites. The 20 top-ranked DMRs are given in Table 4, and all the coordinates and information about the DMRs are given in Additional file 2: Table S10. The most significant DMR was a region on chromosome 10 that contained the promoter and the first exon of the *PPP2R2D* gene, spanning 9 CpG sites. Furthermore, it contained DNase hypersensitive areas and was hypermethylated in W1 as compared to W0 (Additional file 1: Figure S7). Overall, 11 DMRs were situated in the major histocompatibility complex (MHC) on chromosome 6, and five were mapped upstream of imprinted genes (*GNAS*, *GNASAS*, *ASB4*, *HYMAI*, *PLAGL1*, *FAM50B*, *KCNQ1OT1*). Interestingly, we found three DMRs in the *GNAS* gene (GNAS complex locus), a region that has a highly complex imprinted expression pattern. Moreover, four DMRs were located at genes (*VPS52*, *TCIRG1*, and *TRIM39*) that were found to be differentially methylated in the largest schizophrenia (SCZ) whole-blood epigenome-wide association study (EWAS) to date [17].

**Table 4:** The 20 most differentially methylated regions (DMRs) associated with the emergence of dimensional psychopathology

hg19 genomic coordinates	N probes	Mean β FC	Combined *p* value	Gene annotation
chr10:133,747,120-133,748,048	9	0.025	6.44 *×* 10^−04^	*PPP2R2D* (TSS1500; 5′UTR; First Exon; body)
chr6:22,297,336-22,298,146	4	0.042	1.49 *×* 10^−03^	*PRL* (TSS1500; 5′UTR; First Exon)
chr7:158,669,801-158,669,978	5	0.019	1.90 *×* 10^−03^	*WDR60* (body)
chr5:40,908,228-40,908,780	4	0.040	2.25 *×* 10^−03^	*C7* (TSS1500)
chr5:132,200,008-132,200,666	6	0.016	2.27 *×* 10^−03^	*GDF9* (TSS200; First Exon)
chr1:169,702,841-169,703,751	4	0.029	3.41 *×* 10^− 03^	*SELE* (TSS1500; 5′UTR)
chr7:95,114,680-95,115,354	8	0.024	4.84 *×* 10^− 03^	*ASB4* (TSS1500; First Exon)
chr7:54,794,693-54,794,760	3	0.038	4.94 *×* 10^−03^	–
chr6:28,540,442-28,541,039	5	0.021	6.01 *×* 10^−03^	*SCAND3* (body)
chr6:3,129,395-3,129,410	3	0.025	6.11 *×* 10^−03^	*BPHL* (Body); *SNORD6* (TSS200); *SNORA32* (TSS1500); *SNORA25* (TSS1500)
chr6:125,623,465-125,623,573	3	0.035	6.28 *×* 10^−03^	*HDDC2* (TSS1500; TSS200)
chr13:36,421,844-36,421,949	3	0.060	6.33 *×* 10^−03^	*MIR548F5* (body)/*DCLK1* (body)
chr20:39,666,232-39,666,765	3	0.042	6.55 *×* 10^−03^	*TOP1* (body)/*PRO0628* (TSS1500; body)
chr10:682,147-682,486	4	0.037	8.01 *×* 10^−03^	*DIP2C* (body)
chr19:46,999,055-46,999,118	3	0.039	8.13 *×* 10^−03^	*PNMAL2* (5′UTR; First Exon)
chr3:75,263,619-75,263,641	3	0.039	9.35 *×* 10^−03^	–
chr19:55,889,013-55,889,387	4	0.032	0.011	*TMEM190* (body)
chr15:28,272,345-28,272,656	3	0.019	0.012	*OCA2* (body)
chr11:63,136,414-63,137,125	3	0.040	0.012	*SLC22A9* (TSS1500; TSS200)
chr4:76,995,173-76,995,796	4	0.018	0,013	*ART3* (TSS1500; TSS200; 5′UTR)

Wave 0 was used as the reference. Gene annotations were taken from Illumina reference files. *N probes* number of probes included in the differentially methylated region. *Mean β FC* mean β fold change for the region. *Combined p value* Stouffer transformation of the FDRs for individual CpG sites that constituted the DMR. *TSS200* 0–200 bases upstream of the transcriptional start site (TSS). *TSS1500* 200–1500 bases upstream of the TSS

We found that 41 DMRs (45% of the total) were upstream of the TSS of genes. Of these, 11 overlapped with CpG islands (CpGIs), 10 were located at CpG shores (~ 2 kb from CpGIs) and 3 were located at CpG shelves (~ 4 kb from CpGIs). Moreover, it was observed that 10 DMRs (11.1% of the total) were located in intergenic regions, and 52 DMRs (57.8% of the total) were located in gene bodies (coding regions). Of the DMRs in gene bodies, two DMRs overlapped with genes that are highly expressed in the brain (*MOG* and *PDE10A*). We found significant enrichment in the regulation of the sequence-specific DNA-binding transcription factor activity GO biological process (GO: 0051090; FDR = 0.01) (Additional file 2: Table S5).

#### DMRs and their gene expression

**Fig. 4:**
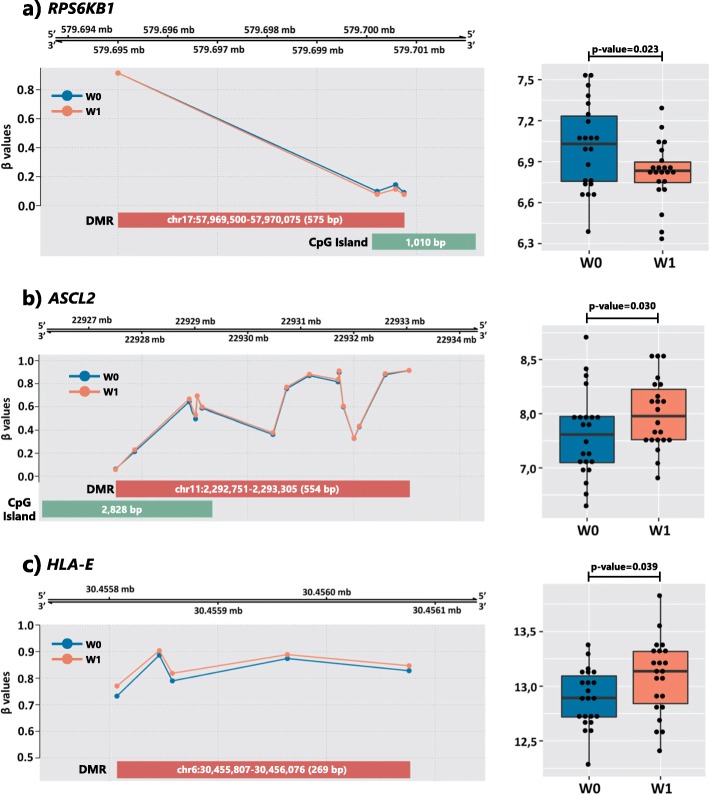
Overview of the differentially methylated regions (DMR) and of their genomic context and box plots of the gene expression levels for the (**a**) RPS6KB1, (**b**) ASCL2, and (**c**) HLA-E genes. The β values of the CpGs that constitute the DMRs are represented by dots. All regions were located 200–1500 bp upstream of the transcriptional start sites. The CpG island track was obtained from the UCSC database. Gene annotation was taken from Illumina reference files according to hg19. Wave 0 was used as the reference. **a** DMR (4 CpG probes; Stouffer transformation p value = 0.021; adjusted p value for the most significant probe = 4.32 × 10−6; mean β fold change = − 0.018) and the gene expression levels (logFC = − 0.194) of RPS6KB1. **b** DMR (17 CpG probes; Stouffer transformation p value = 0.598; adjusted p value for the most significant probe = 3.78 × 10−6; mean β fold change = 0.014) and the gene expression levels (logFC = 0.185) of ASCL2. **c** DMR (5 CpG probes; Stouffer transformation p value = 0.015; adjusted p value for the most significant probe = 1.01 × 10−5; mean β fold change = 0.032) and the gene expression levels (logFC = 0.185) of HLA-E

Of the 86 genes that were overlapped by or were near to the DMRs, 15 had an expression in the peripheral blood that was identified by a gene expression array (Additional file 2: Table S11). We found that *ASCL2*, *RPS6KB1*, and *HLA-E* were differentially expressed in the blood at W0 and W1 (Fig. 4). The DMRs were located 200–1500 bases upstream of the transcriptional start sites of these genes. Two of the DMRs (upstream of *ASCL2* and *RPS6KB1* genes) overlapped with CpGIs, while the other one was located at a CpG shore. Furthermore, the DMR upstream of *RPS6KB1* was located (1) at a DNase hypersensitive region for different cell lines; (2) in a region that included several potential transcription factor binding sites; and (3) in a region enriched for the H3K27Ac histone mark (often found near active regulatory elements) for different cell types.

Regarding the correlation between DNA methylation in the brain and blood for these DMRs, seven and two CpG sites located at the DMRs upstream of *ASCL2* and *HLA-E* genes, respectively, were correlated to the methylation levels in the brain according to the IMAGE-CpG tool [29]. Moreover, a correlation between methylation in the brain and blood for all CpGs located at DMRs upstream of the *RPS6KB1* gene was shown by this tool (Additional file 2: Table S12).

#### DMRs, gene expression, and CBCL score correlation

There were no correlations between the ΔmDMR and ΔmRNA levels (*ASCL2*: *r* = − 0.015; *p* = 0.949; *HLA-E*: *r* = − 0.120; *p* = 0.596; *RPS6KB1*: *r* = 0.194; *p* = 0.388). Moreover, we did not find any correlation between ΔCBCL scores and ΔmDMRs (*ASCL2*: *r* = − 0.083; *p* = 0.715; *HLA-E*: *r* = − 0.224; *p* = 0.316; *RPS6KB1*: *r* = 0.160; *p* = 0.478). However, there was a correlation between the ΔCBCL and *RPS6KB1* ΔmRNA levels (*r* = 0.442; *p* = 0.040). The higher the increase in CBCL scores between W0 and W1, the higher the difference in *RPS6KB1* mRNA levels between the waves (Additional file 1: Figure S8). We did not observe a correlation between the ΔCBCL scores and ΔmRNA levels of the *ASCL2* and *HLA-E* genes (*ASCL2*: *r* = − 0.224; *p* = 0.3171; *HLA-E*: *r* = 0.145; *p* = 0.5198).

## Discussion

Epigenetic studies performed on samples collected before and after the increase of psychopathology have the potential to reveal predictors of mental disorders. We performed a longitudinal epigenome-wide association study on the emergence of dimensional psychopathology during the transition from childhood to adolescence, quantifying the methylation in DNA samples isolated from whole blood. After implementing a methodological framework to reduce the effect of chronological age and the puberty transition on the DNA methylation, we identified 663 DMPs and 90 DMRs associated with the emergence of psychopathology. We observed that 15 DMPs were mapped to genes that were differentially expressed in the blood and that differences in the *RB1CC1* mRNA levels were correlated with differences in DNA methylation between the waves. Furthermore, the variation of *KMT2E* mRNA was correlated with the increase of dimensional psychopathology levels over time. We found that almost half of the total DMRs were located upstream of transcriptional start sites. As promoter regions are generally located in close proximity to the 5′ end of genes [35], these DMRs could be involved in the regulation of gene transcription. Indeed, three genes (*ASCL2*, *RPS6KB1*, and *HLA-E)* were differentially expressed in whole blood of the same individuals, and the mRNA levels of *RPS6KB1* were correlated with the ΔCBCL; this indicated that when the difference in gene expression was greater across time, a greater increase in dimensional psychopathology was observed. Moreover, 4 DMRs were located at genes that were found to be differentially methylated in the blood of chronic patients with SCZ [17], 11 DMRs were situated in MHC loci (the locus most strongly associated with SCZ [17]), and 5 DMRs were located upstream of imprinted genes (many imprinted genes affect behavior). To our knowledge, this is the first human longitudinal study evaluating the association of within-subject changes in DNA methylation with changes in dimensional measurements of psychopathology. We tested the relationship between altered DNA methylation, gene expression, and psychopathology measurements, and our findings suggest the involvement of novel genes in adolescent psychopathology.

### Differentially methylated positions

The differentially methylated positions (DMPs) mapped to genes that were enriched for cell-cell adhesion biological processes and postsynaptic cellular components, suggesting that the differentially methylated genes were involved in cell attachment and that their proteins were located in subcellular structures that are important to brain development. We did not observe an overlap between genes indicated by the 20 top-ranked DMPs and DMRs. The neighboring CpGs of these DMPs are probably not included in the 450 K arrays and were excluded from our analysis. The exclusion of these probes might have influenced the chromosomal regions of the 20 top-ranked DMPs in the DMR analysis.

We observed that 15 genes that overlapped with the DMPs had alterations in their expression levels. Among them were *BCL11A*, *KMT2E*, and *RB1CC1*. Two gene expression probes for *BCL11A* were downregulated in W1 as compared to W0. The corresponding protein product is a transcription factor associated with the mammalian BAF SWI/SNF chromatin remodeling complex in human T cells [36]. A study demonstrated that *BCL11A* haploinsufficiency is implicated in intellectual developmental disorders [37]. Moreover, we found a significant positive correlation between the *KMT2E* ΔmRNA level and ΔCBCL, indicating that there was higher variation in *KMT2E* expression (upregulation in W1 compared to W0) with a higher increase in dimensional psychopathology. *KMT2E* (also known as *MLL5*) is a chromatin regulator expressed throughout the cell cycle and plays a role in hematopoiesis and spermatogenesis [38]. However, an exome sequencing study reported that *KMT2E* is strongly associated with autism spectrum disorder [39]. Finally, we found a significant negative correlation between the cg08517799 ΔDMP and *RB1CC1* ΔmRNA levels, indicating that as CpG methylation increases, the gene expression of *RB1CC1* decreases. *RB1CC1* encodes a tumor-suppressing protein that interacts with signaling pathways to regulate cell growth, proliferation, migration, apoptosis, and autophagy. However, methylation changes in a single CpG are less likely to promote downstream biological consequences. It is more likely that coordinated DNA methylation changes in genomic regions (i.e., DMRs) would have a downstream biological effect.

### Differentially methylated regions

The differentially methylated regions (DMRs) mapped to or near to genes that were enriched for the biological process of the regulation of sequence-specific DNA binding transcription factor activity, suggesting that the differentially methylated genes could be involved in gene expression regulation. In our analysis, the most significant DMR contained a region upstream of the TSS and the first exon of *PPP2R2D* (protein phosphatase 2 regulatory subunit Bdelta)*.* This DMR also contained DNase hypersensitive areas. This region was hypermethylated in W1 as compared to W0, but we did not observe the differential expression of *PPP2R2D* in whole blood between the waves. This gene encodes a regulatory subunit of protein phosphatase 2A that plays a key role in the cell cycle by controlling mitosis entry and exit [40]. *PPP2R2D* is selectively expressed in the mouse hippocampus and is upregulated in the hippocampus of rats during early rapid-eye-movement (REM) sleep after they are exposed to novel objects [41, 42]. A cross-sectional study that aimed to identify the DNA methylation signatures at genes modulating dopamine signaling that are associated with obesity features found that *PPP2R2D* is hypermethylated in the blood of adults with abdominal obesity [43]. Although we performed comparisons within-subjects, and our sample was composed of youths and not adults, we could not determine whether body mass index influenced our results, as this information was unavailable.

We identified three DMRs that mapped to 200–1500 bases upstream of the TSS of genes for which the expression levels in blood were altered between the waves. First, DMR hypermethylation and *ASCL2* gene expression upregulation were observed in W1 compared to W0. *ASCL2* (achaete-scute family bHLH transcription factor 2) is a member of the basic helix-loop-helix family of transcription factors, which are downstream targets of Wnt signaling [44], and it is involved in the determination of neuronal precursors in the peripheral and central nervous systems. Highly expressed in the skin, *ASCL2* is associated with Beckwith-Wiedemann syndrome, which is the most common pediatric overgrowth syndrome [45]. Second, DMR hypermethylation and *HLA-E* gene expression upregulation were observed in W1 as compared to W0. Highly expressed in whole blood, *HLA-E* (major histocompatibility complex, class I, E) is a protein-coding gene that belongs to the non-classical group of MHC-Ib molecules and is mapped to the MHC locus on the short arm of the chromosome 6 [46]. Lastly, DMR hypomethylation and *RPS6KB1* gene expression downregulation were observed in W1 as compared to W0. *RPS6KB1* (ribosomal protein S6 kinase B1) encodes a ribosomal kinase that responds to mTOR (mammalian target of rapamycin) signaling activation to promote protein synthesis, cell growth, and cell proliferation. The highest median expression is in the ovary and has been associated with human cancer [47–49]. Although we did not find a significant correlation between the methylation and expression of these genes, we observed that hypermethylation did not repress their gene expression, suggesting additional mechanisms of gene expression regulation.

Although none of these genes has been directly associated with psychiatric phenotypes, we identified a differentially methylated region of a gene involved in neurogenesis (*ASCL2*). In particular, the DNA methylation levels of almost half of the CpG sites located within this DMR were correlated with the levels in the brain, based on previous analysis [29]. Indeed, studies have shown altered expression of a key gene in Wnt signaling in the hippocampus of patients suffering from neuropsychiatric disorders [50]. Moreover, we identified a differentially methylated and differentially expressed gene located at the MHC region (*HLA-E*), which is the most robustly associated locus in the largest SCZ GWAS and the top-ranked DMR identified in the largest SCZ EWAS to date [17, 51]. Notably, 12% of our results for differentially methylated regions were mapped to the MHC locus, suggesting the involvement of this region in psychiatric symptomatology in adolescence. Furthermore, although there are known to be significant differences in the overall and specific methylation levels between different tissue types [52], few methylation changes were observed in CpG shores between immune system cells [53]. As the DMR that mapped to *HLA-E* is located at a CpG shore, this suggests that methylation of *HLA-E* might not represent a cell type-specific methylation and might be involved in adolescent psychopathology. In addition, we found that a ribosomal kinase that responds to mTOR was differentially methylated and expressed between time points (*RPS6KB1*). As observed in the IMAGE-CpG tool, the DNA methylation levels of all CpGs located at this DMR were correlated with the methylation levels in the brain [29]. Given the rich literature on the role of mTOR in age-related diseases and the evidence of its association with advanced biological aging and mental disorders [54–57], it is striking that the methylation and expression levels of a downstream target of mTOR were altered in a sample that increased in psychopathology over time. Interestingly, we observed that higher expression levels of *RPS6KB* in W1 as compared to W0 were correlated with increased dimensional psychopathology across time. Although we observed hypomethylation of *RPS6KB1*, its expression was downregulated overall between W1 and W0. Thus, more studies are needed to elucidate this relationship.

Another interesting result was the identification of DMRs located at genes that were found to be differentially methylated in the SCZ EWAS [17]. SCZ is a psychiatric disorder with higher heritability, and although it is not yet known if some subjects in our sample will develop SCZ in the future, our results confirm that these genes might be involved in the development of mental disorders, including SCZ. Moreover, we found that some DMRs were located at imprinted genes. Many imprinted genes are expressed in the brain and affect behavior [58]. In adults, imprinted genes are associated with behaviors such as maternal care, sex, feeding, and cognition [59]. In addition, we found three DMRs that mapped to *GNAS*. This locus has a highly complex imprinted expression pattern. It gives rise to maternally, paternally, and biallelically expressed transcripts. In a recent study that investigated the epigenetic profiles of select youth, monozygotic twin pairs who were discordant for anxiety, the differential methylation of loci that were annotated to *GNAS* was associated with anxiety [60]. In addition, we found two DMRs that mapped to *MOG* (myelin oligodendrocyte glycoprotein) and *PDE10A* (phosphodiesterase 10A), genes that are highly expressed in the brain [47]. However, these DMRs were mapped to the gene body, and the DNA methylation patterns between blood and brain are very distinct, particularly in the gene body [61].

### Strengths of the study

First, our study design was longitudinal, and it combined the collection of clinical and DNA methylation data at a baseline and at a 3-year follow-up; this could help to elucidate the temporality of the relationship between the development of psychopathology and methylation changes. Second, we used an epigenome-wide approach to investigate DNA methylation. This approach, without a hypothesis a priori, is crucial for identifying new relevant CpG sites and regions associated with the emergence of dimensional psychopathology in youths. Third, dimensional psychopathology exists on a continuum in the general population, and population-based studies have demonstrated that symptoms, when considered dimensionally, vary with neurobiological features [62, 63], providing further support for the examination of dimensional psychopathology. Indeed, recent initiatives to elucidate the biological causes of mental disorders, such as the Research Domain Criteria (RDoC), focus on the dimensional distribution of several behavioral traits and their neural correlates [64]. Fourth, we studied youths, a group that has had shorter exposure to environmental events that could influence DNA methylation, such as smoking, relative to adults. All 24 of the investigated youths reported that they had never smoked cigarettes or chewed tobacco. Moreover, psychiatric symptomatology during childhood and adolescence predicts persistent mental illness later in life [65]. Fifth, we found DNA methylation changes in blood, a tissue that is accessible via minimally invasive procedures. Sixth, our results seem not to be influenced by medication, as all the DMPs remained significant after the medication was included as a covariable in the analysis.

### Limitations of the study

Despite its longitudinal design and dimensional assessment of psychopathology, the results of this study should be interpreted in light of some limitations. First, methylation patterns are tissue-specific; thus, the methylation differences observed in blood might serve as a marker for phenotypic risk but might not reflect the brain methylome. Nevertheless, our results of most interest showed a correlation between DNA methylation in the brain and blood, as shown in a previous analysis [29], highlighting the possible biological relevance of our findings on the emergence of psychopathology. Second, DNA methylation patterns are also influenced by other factors, such as body mass index and blood cell composition [66]. Because of our sample size, we did not correct our methylation model for estimations of cell composition. Thus, blood cell composition remains a probable confounding factor for the methylation analysis. However, the correspondence of both altered DNA methylation and gene expression is powerful evidence that should be considered even with this limitation. Third, we did not correct gene expression and correlation analyses for multiple comparisons since we chose to be less strict statistically in order to preserve the biological data. Fourth, we did not have a longitudinal control group from the same population to verify whether the DNA methylation differences observed in this study were related to the dynamic nature of DNA methylation, as methylation is the result of complex interactions between genes and the environment that take place over the lifetime of an individual [67]. To overcome these limitations, we employed a DNA methylation marker selection to exclude probes associated with chronological age and the puberty transition in the whole blood of the youths. However, we might have lost DMPs and DMRs associated with the emergence of psychopathology since we had to exclude EPIC probes that were not present in 450 K arrays. Fifth, it is unknown whether the DNA methylation differences observed in this study of blood samples will remain stable over time or will change at the onset of a full-blown psychiatric disorder, since methylation is dynamic throughout development. However, the low stability and high comorbidity patterns of categorical psychopathology in this age range, as assessed by our current classification (e.g., DSM), support our dimensional approach to psychopathology. Sixth, as our analysis focused on the CBCL total score, our results were related to global non-specific psychopathology and not to specific symptoms which could be evaluated by CBCL subscales. Finally, as we performed multiple comparisons in the epigenome-wide approach, we had to correct for multiple testing. Although this correction is necessary, it increases the occurrence of false-negative results.

## Conclusions

In summary, the emergence of dimensional psychopathology appeared concurrently with changes in the patterns of DNA methylation in whole blood cells. Changes in the methylation of single CpGs (DMPs) and regions (DMRs) were observed simultaneously with changes in gene expression levels that were associated with the emergence of psychopathology in youth. Among them, we highlighted those that were annotated to *ASCL2*, which is involved in neurogenesis; to *HLA-E*, which maps to the MHC loci; and to *RPS6KB1*, the gene expression of which was correlated to an increase in dimensional psychopathology. Our data indicate that peripheral blood is a valuable surrogate tissue for the assessment of pathophysiology of behavioral symptoms in youths and could be used to reveal putative peripheral biomarkers. Future epigenetic studies of an independent longitudinal cohort will be required to replicate these findings and to complement this research in order to identify early epigenetic biomarkers for the development of psychopathology.

## Supplementary information

**Additional file 1: **Table S1. Description of CBCL total, internalizing and externalizing scores. Figure S1. Multi-dimensional scaling (MDS) plots colored by BeadChip number, BeadChip position and waves. Figure S2. PC1 x PC2 plot from principal components analysis generated from SNParray data. Figure S3. Line plot of chronological age between Wave 0 and Wave 1 for the 24 HRC individuals, grouped by gender. Figure S4. Scatter plot of the variation of cg08517799 DNA methylation values and the variation of *RB1CC1* mRNA levels (rp = − 0.580; *p* = 0.005). Figure S5. Scatter plot of the variation of total score of CBCL and the variation of *KMT2E* mRNA levels (rp = 0.436; *p* = 0.042). Figure S6. Scatter plot of the variation of total score of CBCL and the variation of *FAM217B* mRNA levels (rp = 0.475; *p* = 0.025). Figure S7. Overview of the most significant differentially methylated region (DMR) and of its genomic context. Figure S8. Scatter plot of the variation of total score of CBCL and the variation of *RPS6KB1* mRNA levels (rp = 0.442; *p* = 0.040). Figure S9. Plot of bisulfite conversion median value for each sample from High-Risk Cohort (HRC). Figure S10. Plot of bisulfite conversion median value for each sample from Philadelphia Neurodevelopmental Cohort (PNC). Figure S11. Error bar plot comparing blood cell type estimations (CD19+ B cells, CD4+ T cells, CD8+ T cells, granulocytes, CD14+ monocytes and CD56+ natural killer cells) between Wave 0 and Wave 1. Figure S12. Principal components analysis plot of the 24 HRC participants and of the population from 1000 genomes.

**Additional file 2:** Table S2. The 100 top-ranked differentially methylated CpGs associated with dimensional psychopathology using all the CpG sites of the EPIC array and included covariates for batch effects. Table S3. The differentially methylated CpGs associated with chronological age in the Philadelphia Neurodevelopmental Cohort (PNC). Table S4. The differentially methylated CpGs (DMPs) associated with the emergence of dimensional psychopathology using batch effects as covariates. Table S5. Overview of significant results from the enrichment analysis obtained using all genes for which the DMPs/DMRs were mapped to or near to the genes. Table S6. Statistics, beta fold change and adjusted *p*-values for the 663 differentially methylated CpGs (DMPs) associated with the emergence of dimensional psychopathology using batch effects and medication as covariates. Table S7. Gene expression results of genes for which the DMPs were mapped to or near to the genes. Table S8. DNA methylation correlation between brain and blood for DMPs mapped or near to differentially expressed genes. Correlation results are from IMAGE-CpG (Braun, P et al., 2019). Table S9. Information about correlations between: a) the variation of the DNA methylation of the differentially methylated positions (ΔDNAm) and the variation of the expression of genes that were differentially expressed in whole blood (ΔmRNA); b) the variation of the total score of CBCL (ΔCBCL) and ΔDNAm; and c) ΔCBCL and ΔmRNA. The variations were calculated subtracting wave 1 (W1) values from wave 0 (W0) values. Table S10. Differentially methylated regions from DMRCate analysis. Table S11. Gene expression results of genes for which the DMRs were mapped to or near to the genes. Table S12. DNA methytion correlation between brain and blood for CpG site from DMRs mapped or near to differentially expressed genes. Correlation results are from IMAGE-CpG (Braun, P et al., 2019).

## Data Availability

The HRC DNA methylation data analyzed during the current study are available from the corresponding author upon reasonable request. In the near future, the DNA methylation data of HRC will be available in the ArrayExpress Archive of Functional Genomics Data (European Bioinformatics Institute).

## Abbreviations

BH: Benjamini–Hochberg
CBCL: Child Behavior Checklist
CpGIs: CpG islands
DAWBA: Development and Well-Being Assessment
DMPs: Differentially methylated positions
DMRs: Differentially methylated regions
EWAS: Epigenome-wide association study
FDR: False discovery rate
GO: Gene ontology
GSEA: Gene Set Enrichment Analysis
GWAS: Genome-wide association studies
HRC: High-risk cohort
KEGG: Kyoto Encyclopedia of Genes and Genomes
MHC: Major histocompatibility complex
PC1: First principal component derived from PCA
PC2: Second principal component derived from PCA
PCA: Principal components analysis
PCs: Principal components derived from PCA
PNC: Philadelphia Neurodevelopmental Cohort
QC: Quality control
ROC: Receiver operating characteristic
SCZ: Schizophrenia
TSS: Transcriptional start site
W0: Wave 0, representing baseline
W1: Wave 1, representing 3 years of follow-up
WEB-Gestalt: WEB-based GEne SeT AnaLysis Toolkit
