# Supplementary material for: Detecting multiple differentially methylated CpG sites and regions related to dimensional psychopathology in youths
Source: Clin Epigenetics. 2019 Oct 21;11:146. doi: 10.1186/s13148-019-0740-z (PMC6805541; doi:10.1186/s13148-019-0740-z)
Supplement: Supplementary file 1 — Additional file 1: Table S1. Description of CBCL total, internalizing and externalizing scores. Figure S1. Multi-dimensional scaling (MDS) plots colored by BeadChip number, BeadChip position and waves. Figure S2. PC1 x PC2 plot from principal components analysis generated from SNParray data. Figure S3. Line plot of chronological age between Wave 0 and Wave 1 for the 24 HRC individuals, grouped by gender. Figure S4. Scatter plot of the variation of cg08517799 DNA methylation values and the variation of RB1CC1 mRNA levels (rp = − 0.580; p = 0.005). Figure S5. Scatter plot of the variation of total score of CBCL and the variation of KMT2E mRNA levels (rp = 0.436; p = 0.042). Figure S6. Scatter plot of the variation of total score of CBCL and the variation of FAM217B mRNA levels (rp = 0.475; p = 0.025). Figure S7. Overview of the most significant differentially methylated region (DMR) and of its genomic context. Figure S8. Scatter plot of the variation of total score of CBCL and the variation of RPS6KB1 mRNA levels (rp = 0.442; p = 0.040). Figure S9. Plot of bisulfite conversion median value for each sample from High-Risk Cohort (HRC). Figure S10. Plot of bisulfite conversion median value for each sample from Philadelphia Neurodevelopmental Cohort (PNC). Figure S11. Error bar plot comparing blood cell type estimations (CD19+ B cells, CD4+ T cells, CD8+ T cells, granulocytes, CD14+ monocytes and CD56+ natural killer cells) between Wave 0 and Wave 1. Figure S12. Principal components analysis plot of the 24 HRC participants and of the population from 1000 genomes. [file 13148_2019_740_MOESM1_ESM.docx]

# Detecting multiple differentially methylated CpG sites and regions related to dimensional psychopathology in youths

Leticia M. Spindola ^a,b,c^, Marcos L. Santoro ^a,b,c^, Pedro M. Pan ^b,c^, Vanessa K. Ota ^a,b^, Gabriela Xavier ^a,b^, Carolina M. Carvalho ^a,b,c^, Fernanda Talarico ^a,b^, Patrick Sleiman ^f^, Michael March ^f^, Renata Pellegrino ^f^, Elisa Brietzke ^c^, Rodrigo Grassi-Oliveira ^g^, Jair J. Mari ^b,c^, Ary Gadelha ^b,c^, Euripedes C. Miguel ^h^, Luis A. Rohde ^i^, Rodrigo A. Bressan ^b,c^, Diego R. Mazzotti ^e^, João R. Sato ^d^ , Giovanni A. Salum ^i^ , Hakon Hakonarson ^f^, Sintia I. Belangero ^a,b,c^*

a Genetics Division, Department of Morphology and Genetics, Universidade Federal de São Paulo (UNIFESP), São Paulo, Brazil

b LiNC - Interdisciplinary Laboratory of Clinical Neurosciences, UNIFESP, São Paulo, Brazil

c Department of Psychiatry, UNIFESP, São Paulo, Brazil

d Center of Mathematics, Computing and Cognition, Universidade Federal do ABC, Santo André, Brazil

e Center for Sleep and Circadian Neurobiology, University of Pennsylvania, Philadelphia, United States

f Center for Applied Genomics, The Children's Hospital of Philadelphia, Philadelphia, United States

g Brain Institute, Pontifícia Universidade Católica do Rio Grande do Sul (PUCRS), Porto Alegre, Brazil

h Department of Psychiatry, Faculdade de Medicina da Universidade de São Paulo (FMUSP), São Paulo, Brazil

i Department of Psychiatry, Hospital de Clínicas de Porto Alegre, Universidade Federal do Rio Grande do Sul (UFRGS), Porto Alegre, Brazil

* Corresponding author:

Sintia Iole Belangero. Genetics Division, Federal University of Sao Paulo (UNIFESP). Address: Rua Botucatu 740, Ed. Leitão da Cunha, Vila Clementino, Sao Paulo – SP, Brazil. Email: sinbelangero@gmail.com

# Study Population

This study is part of a large, community school-based survey that combines standardized evaluation with a neurodevelopmental approach. The High-Risk Cohort (HRC) for Psychiatric Disorders aims to map neurodevelopmental trajectories in typical development and in common mental illnesses [[1](#_ENREF_1)].

## **Wave 0 (W0) – Baseline**

The baseline assessment (wave 0) was performed in 4 phases, as previously described: 1) screening; 2) household parent interview; 3) child cognitive evaluation (school interview); and 4) neuroimaging and peripheral-biomarker testing. The study population in the screening phase comprised 6–12-year-old students from 22 public schools in Porto Alegre (PoA) and 35 schools in São Paulo (SP), Brazil. Inclusion criteria were as follows: registered for school by a biological parent capable of providing consent and information regarding the child’s behavior; age between 6 and 12 years; and enrolled in the same school during the year. For screening, 9,937 informant interviews (88% with biological mothers) on the Family History Survey (FHS) were conducted [[2](#_ENREF_2)]. From this pool, we selected 2 subgroups: a community random group and a high-risk of psychiatric disorder stratum. From the FHS, we extracted an index of family load that expressed the percentage of family members who screened positive for the psychiatric disorders evaluated. The high-risk group was determined based on this index.

From 1,315 children selected for the random stratum, 958 (73%) completed the household parent interview phase. From the 2,050 children selected for the high-risk stratum, 1,554 (76%) participated in the study. The entire sample composed of the random and high-risk groups included 2,512 participants. The Development and Well-Being Assessment (DAWBA) structured diagnostic interview was used to evaluate psychiatric diagnosis according to the Diagnostic and Statistical Manual of Mental Disorders, Fourth Edition (DSM-IV) [[3](#_ENREF_3)].

From the total cohort of 2,512 participants, 1,004 children were invited to participate in neuroimaging and peripheral-biomarker testing; 751 children (and their parents/guardians) accepted the invitation to participate in MRI scanning, and 625 agreed to blood-sample collection. We collected blood in one EDTA tube (Becton Dickinson (BD), Franklin Lakes, NJ) for DNA analysis; one PAXgene® RNA tube (PreAnalytix, Hombrechtikon, Switzerland) for RNA analysis; and one Gel SST II Advance tube (BD) for protein analysis. On the same day as the blood collection, the parents/caregivers also completed the Child Behavior Checklist (CBCL), a parent-report questionnaire based on which each child was rated for various behavioral and emotional problems [[4](#_ENREF_4)].

## **Wave 1 (W1) – 3 years of follow-up**

Three years later, we contacted the parents to ask them to participate in the HRC follow-up. The first follow-up evaluation included a household visit by a lay interviewer, who interviewed the parents or main caregivers of study subjects. In a second household visit, certified psychologists interviewed the adolescents. In 10.2% (N=255) of cases, we were unable to contact any family member using all available information. Strategies to contact these individuals included telephoning family members, calling at several different times of day, searching school registries, attempting contact by mail, and visiting the address where the baseline evaluation occurred. Another 9.8% (N=246) of families declined to participate in the follow-up evaluation. The remaining sample consisted of 2,010 participants, comprising 80.05% of the baseline sample. Higher maternal education (χ^2^=14.07; p<0.001) and socioeconomic status (χ^2^=6.24; p<0.05), living in PoA (χ^2^=4.57; p<0.05), and having a child who met criteria for an anxiety disorder at baseline (χ^2^=9.75; p<0.01) were associated with a higher likelihood of successful follow-up.

From the total of 2,010 participants, 493 youths (and their parents/guardians) participated in MRI scanning, and 479 agreed to blood-sample collection. We collected blood in two EDTA tube (BD) for DNA analysis; two PAXgene® RNA tube (PreAnalytix) for RNA analysis; and two Gel SST II Advance tube (BD) for protein analysis. On the same day as the blood collection, the parents/caregivers also completed the CBCL.

# Participant Selection

From the pool of subjects with good-quality blood samples available for both waves, we selected subjects who met the following four criteria: 1) they lived in São Paulo (to exclude site effects) at both time-points; 2) they did not fulfill DSM-IV criteria for any mental disorder in the DAWBA and presented with low levels of dimensional psychopathology at the baseline (CBCL total score < 30.5 at W0); 3) they presented with high levels of dimensional psychopathology at the 3-year follow-up (CBCL total score ≥ 30.5 at W1); and 4) they presented with important changes in the dimensional psychopathology levels between assessments (ΔCBCL = CBCL_W1_ – CBCL_W0_ > 15).

This classification was chosen based on a previous study by our group (Ota&Santoro et al., in preparation). We aimed to classify the participants into a group that increased psychiatric symptomatology severity between the waves to investigate gene expression changes in the blood associated with different trajectories of mental disorders development. For choosing a point to classify the participant into “low psychiatric symptoms” or “high psychiatric symptoms”, it was performed a Receiver Operating Characteristic (ROC) curve analysis using the CBCL total score as a predictor of mental disorder according to the DAWBA. Thus, the point of 30.5 on the CBCL represented the best cut-off to detect mental disorders in the sample (sensitivity=75.6%, specificity=73.7% and Younden's J=0.019). As we were interested in a group that has increased symptomatology severity over time and considering that the CBCL scores do not increase over time in our population (r_p_=0.071; p-value=0.074), the CBCL variation between W1 and W0 had to be higher than 15 points, since this represented a half standard deviation of the CBCL score distribution.

# Validation literature from CBCL

The CBCL is a widely used inventory that provides parent-report information on a wide array of behavioral problems in young, composed by 120 items rated as Not True (0), Somewhat or Sometimes True (1), or Very True or Often True (2). Therefore, CBCL total score could range from 0 to 240.

The validation literature from CBCL, which is presented in ASEBA manual [[4](#_ENREF_4)] and was derived from a US epidemiological national sample, showed that average CBCL total score from clinically referred sample ranges from 40 to 50 points, according to age (6 to 18 years old), whereas the nonreferred sample ranges from 17 to 22. Therefore, clinically important average levels are above 30 and typical nonreferred average levels are below 22, supporting our empirically ROC derived 30.5-point cut-off.

Although we set that ΔCBCL>15, we observed that mean change between baseline and follow-up was significantly higher than 15 points (Table S1). This variation could possibly show a status change between nonreferred to referred mean values according to ASEBA manual.

We also verified the participant's variability on CBCL internalizing and externalizing subscales (Table S1), as they comprise a well-established 2-factor structure of childhood and adolescence psychopathology. We observed that these subscales showed a mean change between baseline and follow-up lower than the CBCL total problem.

Table S1: Description of CBCL total, internalizing and externalizing scores.

| **Variables** |  | **W0** | **W1** | **Delta**  **(W1-W0)** |
| --- | --- | --- | --- | --- |
| **Total CBCL** | Mean | 18.79 | 48.08 | 29.29 |
|  | SD | 7.90 | 10.82 | 9.34 |
|  | Median | 21.00 | 47.50 | 26.50 |
|  | Min | 0 | 31 | 16 |
|  | Max | 30 | 79 | 52 |
| **Internalizing CBCL** | Mean | 6.71 | 17.67 | 10.96 |
|  | SD | 4.38 | 7.51 | 6.98 |
|  | Median | 6.5 | 18.0 | 9.50 |
|  | Min | 0 | 6 | 0 |
|  | Max | 17 | 33 | 33 |
| **Externalizing CBCL** | Mean | 1.54 | 3.29 | 1.75 |
|  | SD | 1.32 | 2.26 | 2.07 |
|  | Median | 2 | 4 | 2.50 |
|  | Min | 0 | 0 | -3 |
|  | Max | 5 | 7 | 6 |

W0: wave 0 represented the baseline; W1: wave 1 represented a 3-year follow-up; SD: standard deviation; CBCL: Child Behavior Checklist.

Additional supplementary tables (Tables S2-S11) are available separately in the .xlsx supplementary file.

# Genome-wide quantification of DNA methylation – Quality Control

The raw intensity files (idat) generated by the Infinium MethylationEPIC BeadChips (“EPIC array” – Illumina, San Diego, CA) were imported into the R programming environment using the read.metharray.exp() function from minfi package [[5](#_ENREF_5)]. The methylation level at each CpG site was calculated as a beta value [β = methylated intensity / (methylated intensity + unmethylated intensity)], that varies from zero (no methylation) to one (complete methylation). Quality control (QC) of the HRC data was verified using different R packages. Before QC, we converted the EPIC array (866,238 probes) into a 450K virtual array (452,832 probes) in order to compare it to Philadelphia Neurodevelopmental Cohort (PNC) [[6](#_ENREF_6)] samples and to Almstrup et al. (2016) study [[7](#_ENREF_7)]. The QC was adapted from Hannon et al. (2016) [[8](#_ENREF_8)] and Maksimovic et al. (2016) [[9](#_ENREF_9)], including the following steps:

1) to check if there was batch effect using multidimensional scaling (MDS) plots (Figure S1);

2) to check the bisulfite conversion using the bscon() function from wateRmelon package (v1.24.0) (Figure S9), excluding samples with median <85;

3) to check the median intensity in both the methylated and unmethylated channels;

4) by using the tissue prediction tool from the Epigenetic Clock software (https://dnamage.genetics.ucla.edu/) [[10](#_ENREF_10)], to check if all predicted tissue were from blood origin (whole blood or blood PBMC) (all predicted tissue were from blood origin);

5) to check the reported sex using MDS plot of probes on sexual chromosomes (the reported sex was correct);

6) to check if the pairs W0-W1 were from the sample individual using the 65 SNP probes (all pairs W0-W1 were correct);

7) to check whether there were samples with mean detection p-value>0.05 (no sample with mean detection p-value>0.05);

8) to filter out probes that have failed in one or more samples based on detection p-value (detection p-value>0.01) (8,477 probes excluded, remained 444,355);

9) to remove probes on the sex chromosomes (9,909 probes excluded, remained 434,446);

10) to remove probes with SNPs at CpG sites using minfi dropLociWithSnps() function (14,099 probes excluded, remained 420,347);

11) to exclude cross reactive probes based on Chen et al. (2013) [[11](#_ENREF_11)] (24,495 probes excluded, remained 395,852);

12) to filter probes using rmSNPandCH() function of DMRcate package [[12](#_ENREF_12)] (6,190 probes excluded, remained 389,662).

Normalization of DNA methylation data was performed used the preprocessFunnorm() function in the minfi package. None of the HRC samples were excluded during QC, which totaled 48 biological samples from 24 individuals.

For PNC samples, raw intensity files generated by Infinium HumanMethylation450 BeadChips (450K arrays – Illumina) were imported in R, and QC was performed as for the HRC samples. Two samples showed the bisulfite conversion median value lower than 85.00 and were excluded from further analysis (Figure S10). One sample showed lower methylated and unmethylated signal intensities (median < 1,500) and was also excluded. Altogether, we excluded 3 PNC samples during QC and analyzed DNA methylation data from 137 individuals. Linear regression models were used to identify the probes associated with age (independent variable) without including any covariates.

DMR plots were drawn with the Gviz package and the other plots were drawn with the ggplot2 package, both in RStudio (v1.1.453).

# Checking for confounders: cell composition and race

Human blood cells have a different pattern of methylation depending on the proportion of cell type [[13](#_ENREF_13)]. Therefore, different proportion of cell types within blood may represent a major source of variability for methylation analyses [[13](#_ENREF_13), [14](#_ENREF_14)].

We used the methylation data for estimation of blood cell composition using the estimateCellCounts() function from minfi package; the flowSorted.Blood.450k was used as reference panel. We estimated the composition of B cells, CD4 T cells, CD8 T cells, granulocytes, monocytes and natural killer cells for each sample and verified whether the blood cell composition estimates were different between the W0 and W1 using gee() function from gee package. It was used gaussian distribution and independence as expected autocorrelation structure.

The result of blood cell composition estimation is summarized in Figure S11. On average, granulocytes represented 58% of the whole blood content, while the five other types represented less than 15%. Those results agree with the values reported by Jaffe & Irizarry (2014) [[14](#_ENREF_14)], which aggregate the data of 1098 samples from five independent studies on whole blood. We did not find significant differences of cell composition estimates between W0 and W1. As our sample size is small and the number of variables in the model could lead to an overfitting problem, and because of the similarity of blood cell composition between waves, the estimates were not added as covariates to the regression models.

Moreover, we checked whether all participants had similar genetic ancestry, by verifying whether the first two PCs generated from the SNParray data were correlated with the first ten PCs from the methylation data, and we did not find any significant correlation. We also verified whether all the HRC participants have a similar ancestry comparing them with the samples from 1000 genomes (Figure S12). They seem to be very admixture compared to population from 1000 genomes Project [[15](#_ENREF_15)], but not very different from one each other. Therefore, the genetic ancestry was not added as covariates to the regression models because of the similarities of the genetic ancestry among participants.

# Metric Table - WEB-based GEne SeT AnaLysis Toolkit (WEB-Gestalt)

The metric table uploaded into WEB-Gestalt was done as follows: 1) to do a vector with the signs of beta fold change (1 if the beta fold change is positive; -1 if the beta fold change is negative); 2) to do a vector with -log10 values of adjusted p-values; 3) to divide -log10 of adjusted p-values by the signs of Beta fold change.

# Figure S1: Multi-dimensional scaling (MDS) plots colored by BeadChip number, BeadChip position and waves. The variability among the samples was more related to the pairs W0-W1 than to the BeadChip number or position.

| 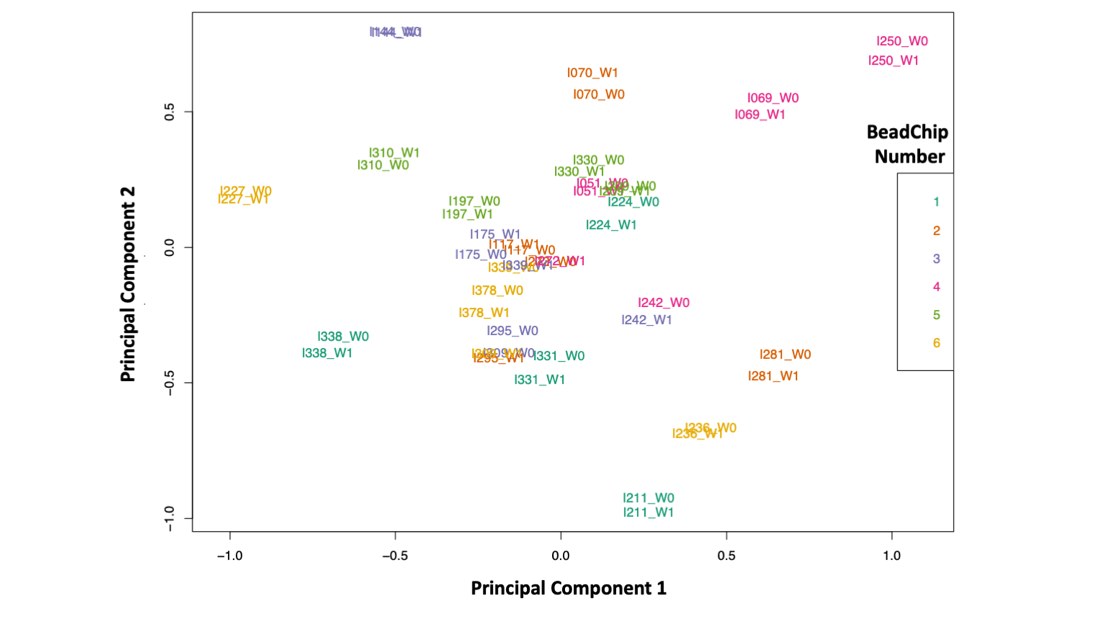 | 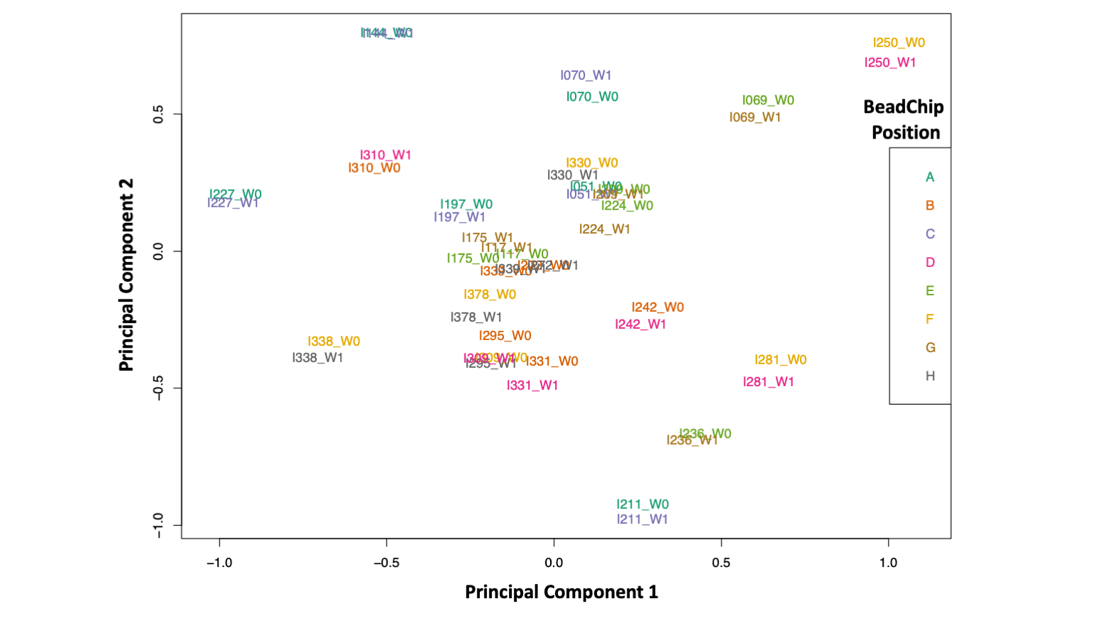 |
| --- | --- |
| 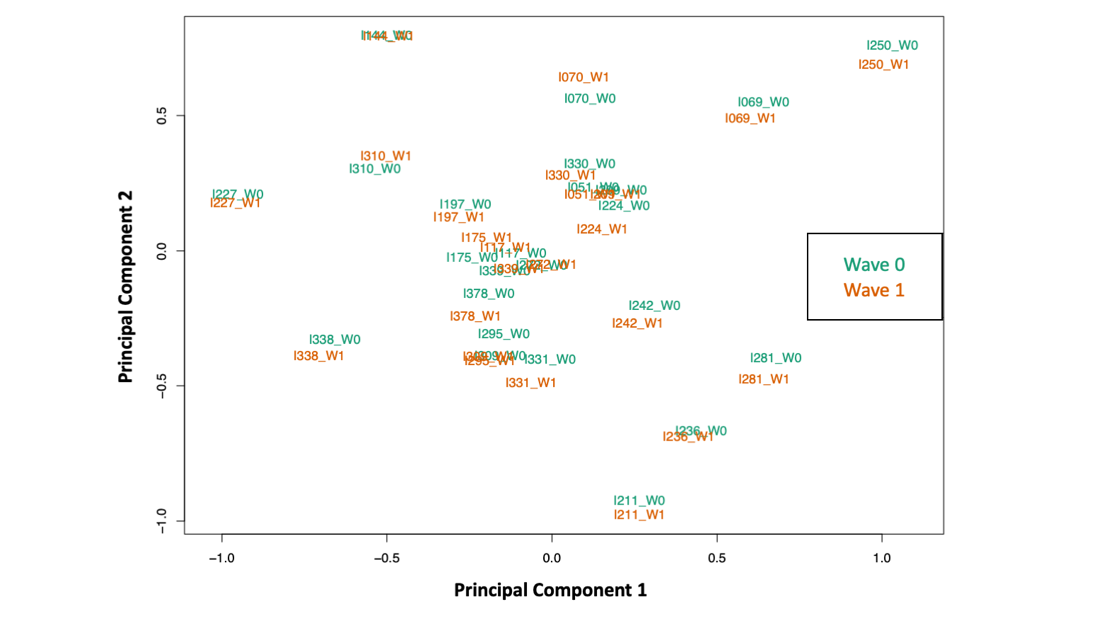 | |

# Figure S2: PC1 x PC2 plot from principal components analysis generated from SNParray data. To select healthy controls from Philadelphia Neurodevelopmental Cohort (PNC, n=870 individuals) with similar genetic ancestry compared to the High-Risk Cohort (HRC), selected individuals who were within the ranges: - 0.035<PC1<0.028 and PC2>-0.1. These ranges were chosen based on a visual inspection. After applied this criteria, 195 individuals from the PNC were selected, but only 140 individuals had the age at the date of the blood collection within the age range of the HRC samples (from 7- to 17-years-old).

# Figure S3: Line plot of chronological age between Wave 0 and Wave 1 for the 24 HRC individuals, grouped by gender.

# Figure S4: Scatter plot of the variation of cg08517799 DNA methylation values and the variation of *RB1CC1* mRNA levels (rp=-0.580; p=0.005).


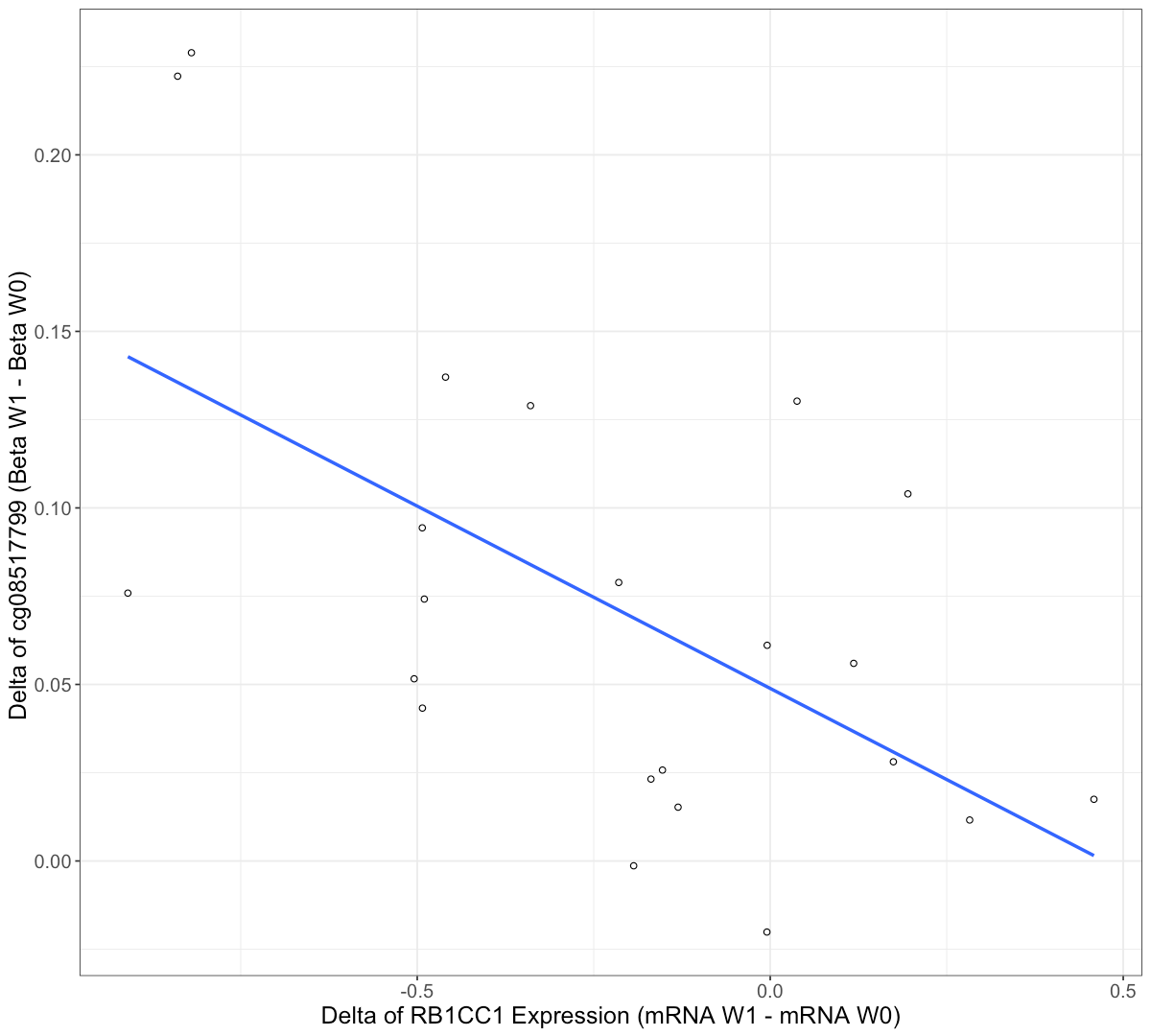


# Figure S5: Scatter plot of the variation of total score of CBCL and the variation of *KMT2E* mRNA levels (rp=0.436; p=0.042).


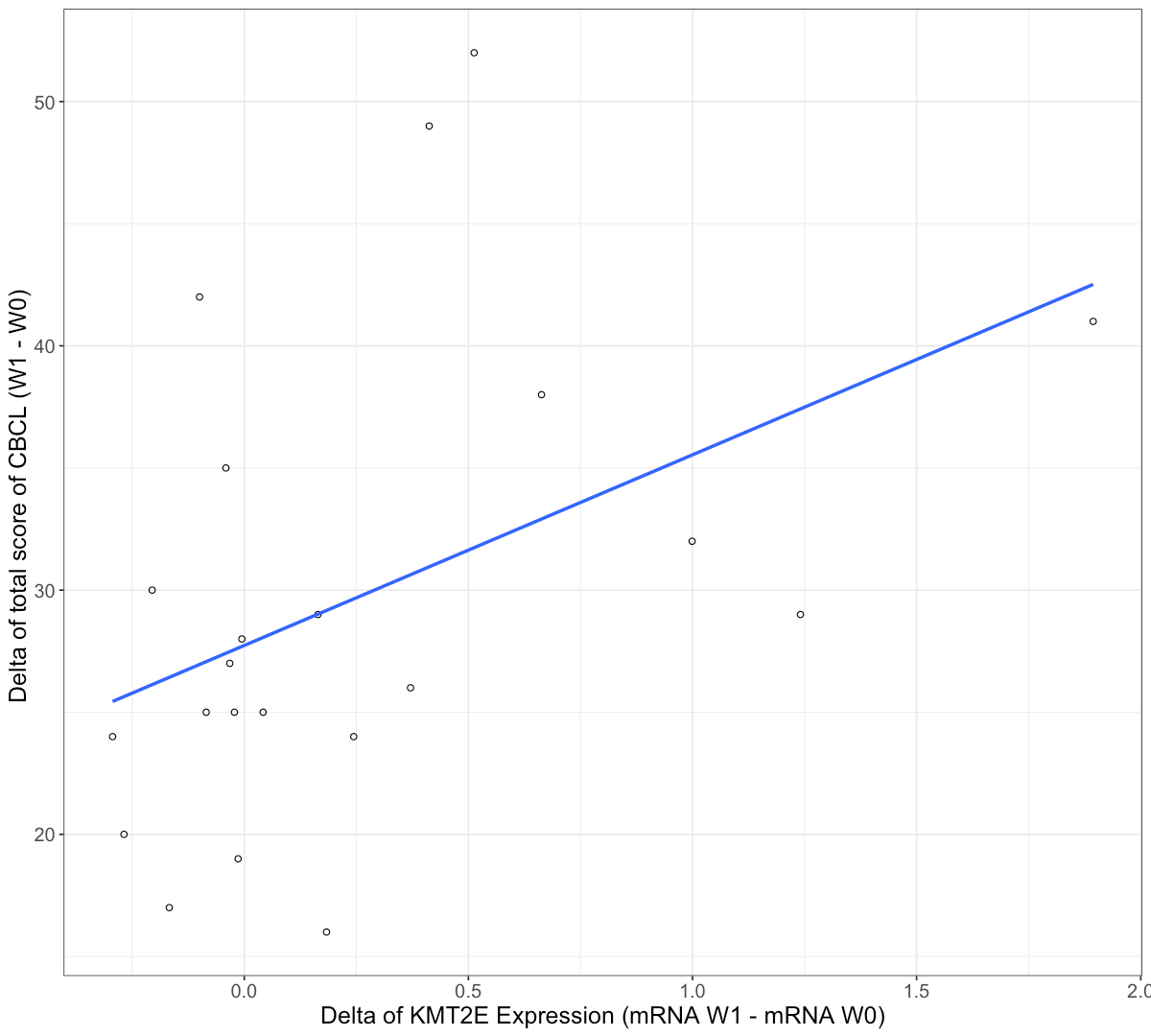


# Figure S6: Scatter plot of the variation of total score of CBCL and the variation of *FAM217B* mRNA levels (rp=0.475; p=0.025).


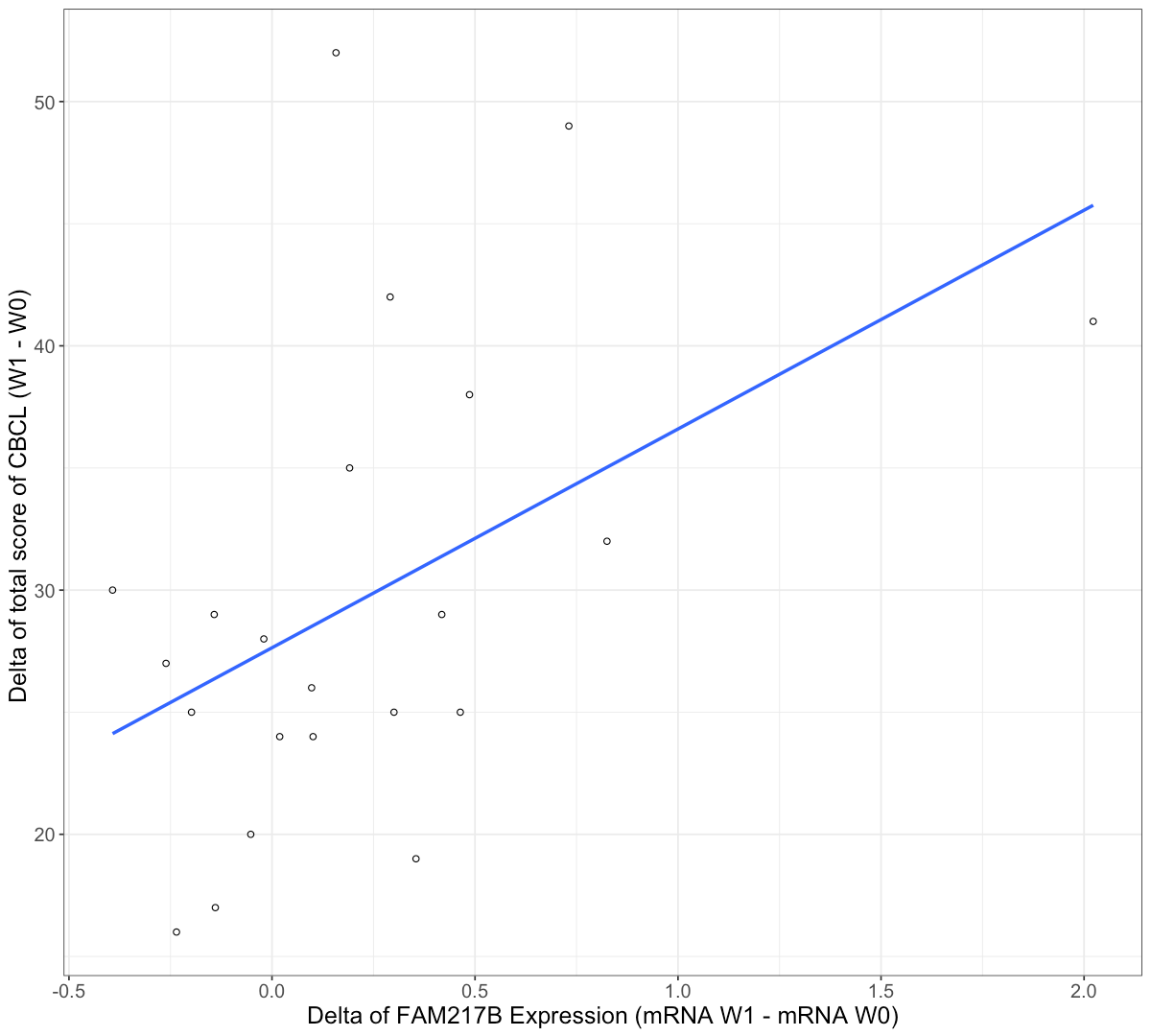


# Figure S7: Overview of the most significant differentially methylated region (DMR) and of its genomic context. The β values of the CpGs that constitute the DMR are represented by dots. This DMR was composed of 9 CpG probes and was found on chromosome 10, that that contains the promoter and the first exon of the *PPP2R2D* gene. Furthermore, it contains DNase hypersensitive areas and was hypermethylated in W1 compared to W0. The CpG island and DNase1 clusters tracks were obtained from the UCSC database. Gene annotation was taken from Illumina reference files according to hg19.

# Figure S8: Scatter plot of the variation of total score of CBCL and the variation of *RPS6KB1* mRNA levels (rp=0.442; p=0.040).


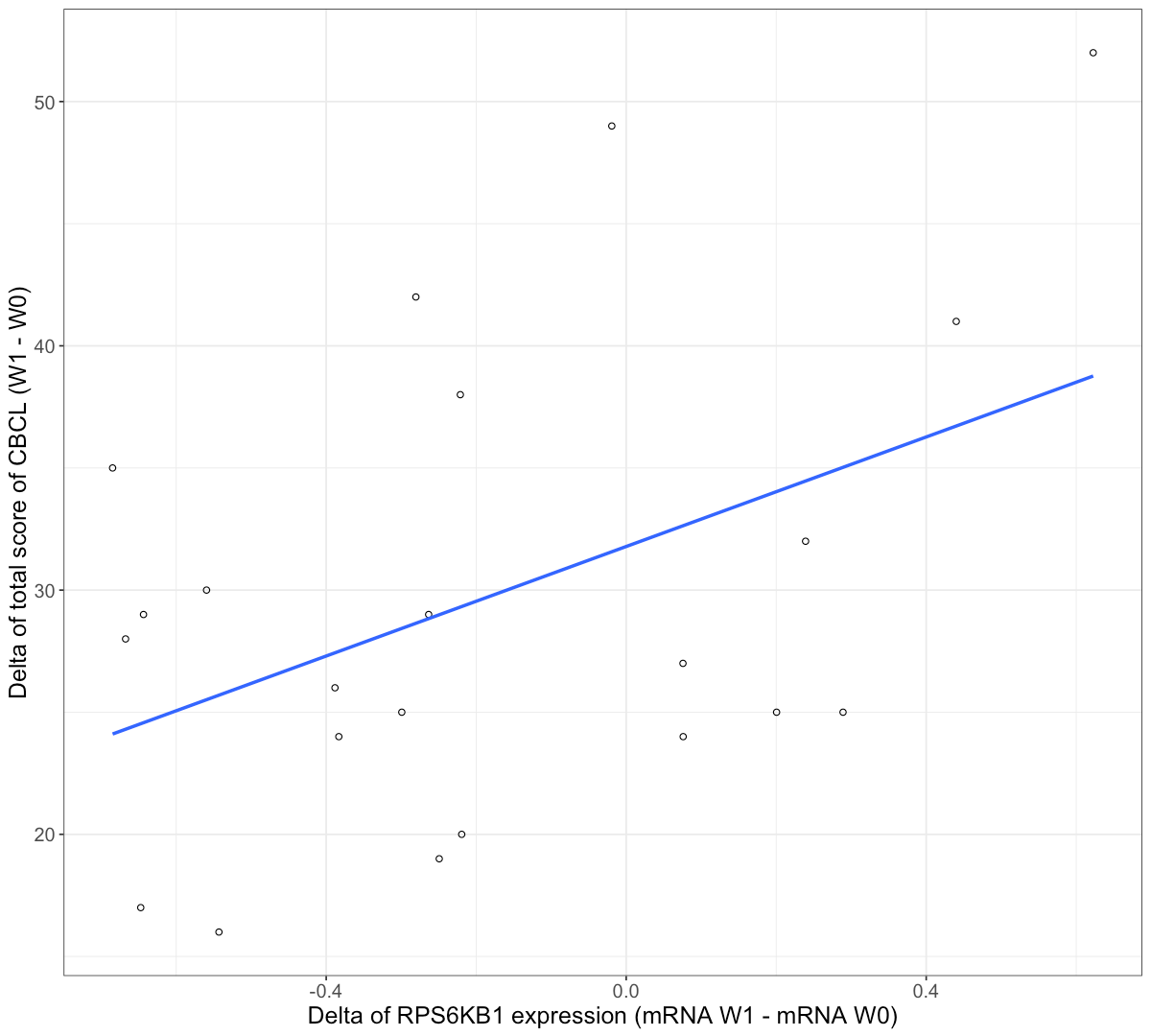


# Figure S9: Plot of bisulfite conversion median value for each sample from High-Risk Cohort (HRC).


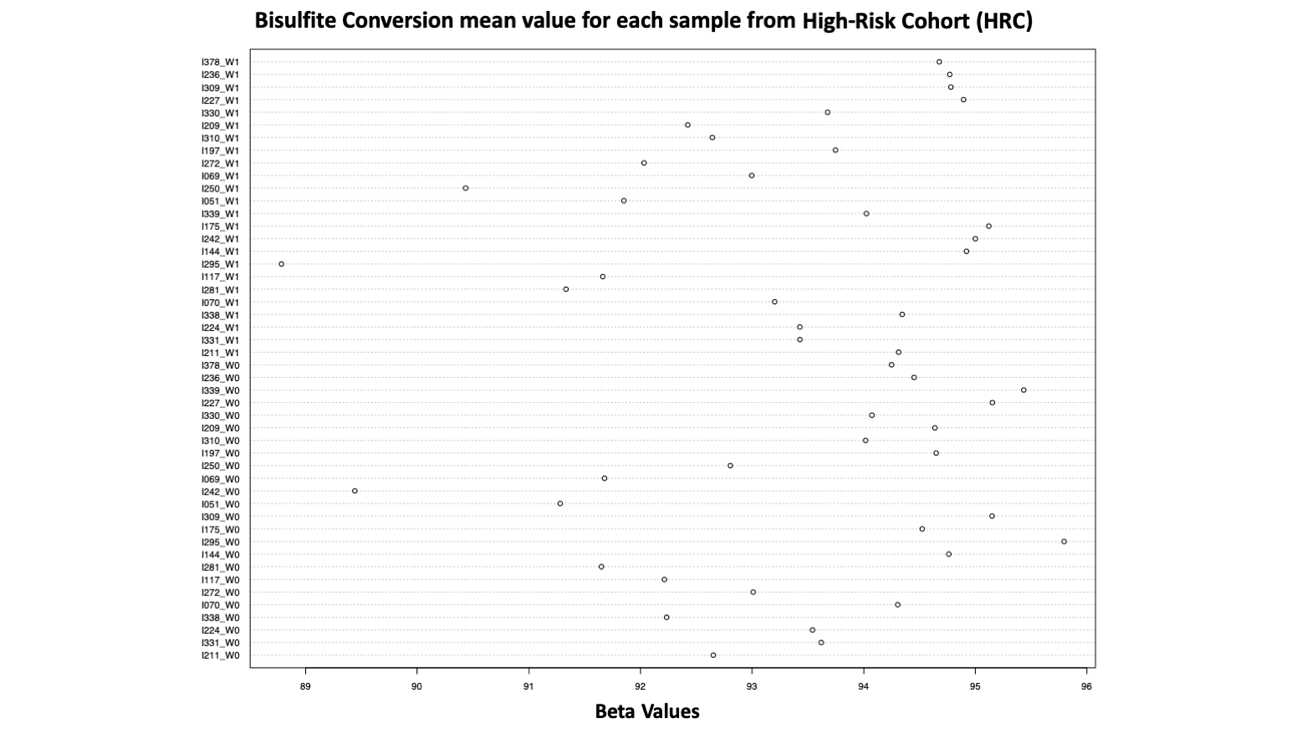


# Figure S10: Plot of bisulfite conversion median value for each sample from Philadelphia Neurodevelopmental Cohort (PNC). The median value of sample 52 (indicated by red arrow) was 85.03.


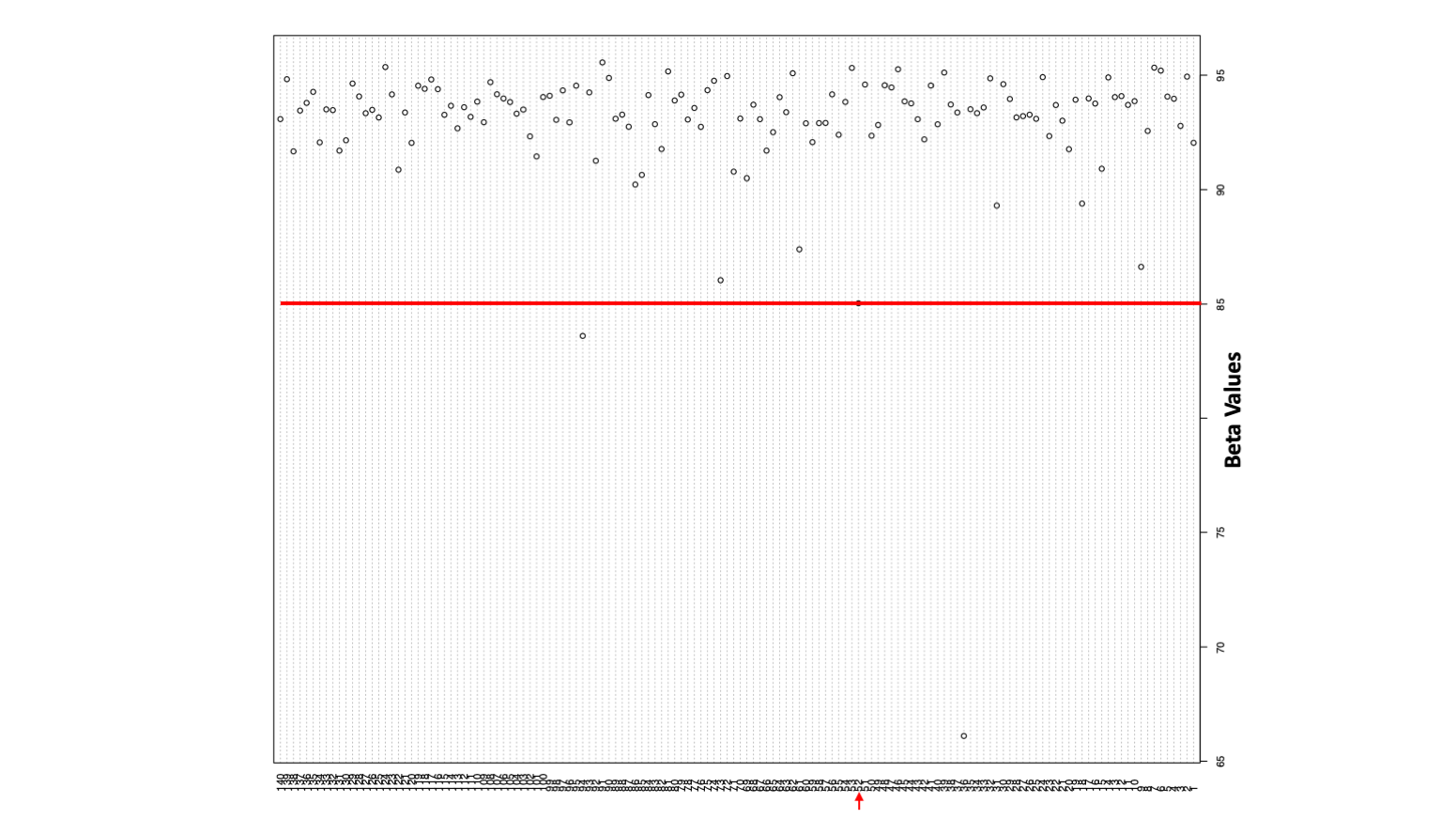


# Figure S11: Error bar plot comparing blood cell type estimations (CD19+ B cells, CD4+ T cells, CD8+ T cells, granulocytes, CD14+ monocytes and CD56+ natural killer cells) between Wave 0 and Wave 1. On average, granulocytes represented 58% of the whole blood content, while the five other types represented less than 15%. Those results agree with the values reported by Jaffe & Irizarry (2014) [[14](#_ENREF_14)], which aggregate the data of 1098 samples from five independent studies on whole blood.

# Figure S12: Principal components analysis plot of the 24 HRC participants and of the population from 1000 genomes. The HRC participants were represented as “My sample” in the plot.


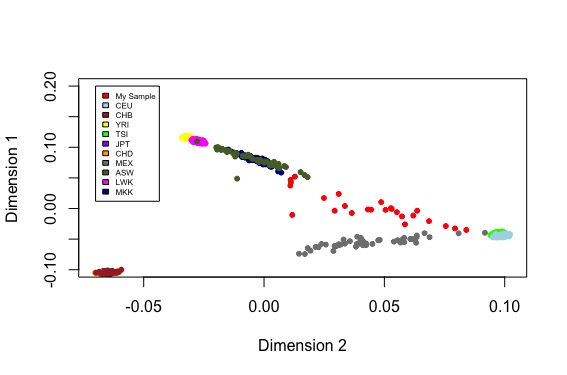


# REFERENCES

1. Salum, G.A., et al., *High risk cohort study for psychiatric disorders in childhood: rationale, design, methods and preliminary results.* Int J Methods Psychiatr Res, 2015. **24**(1): p. 58-73.

2. Weissman, M.M., et al., *Brief screening for family psychiatric history: the family history screen.* Arch Gen Psychiatry, 2000. **57**(7): p. 675-82.

3. Goodman, R., et al., *The Development and Well-Being Assessment: description and initial validation of an integrated assessment of child and adolescent psychopathology.* J Child Psychol Psychiatry, 2000. **41**(5): p. 645-55.

4. Achenbach, T.M. and L.A. Rescorla, *Manual for the ASEBA School-Age Forms & Profiles*. 2001, Burlington, VT: Research Center for Children, Youth, & Families, University of Vermont.

5. Aryee, M.J., et al., *Minfi: a flexible and comprehensive Bioconductor package for the analysis of Infinium DNA methylation microarrays.* Bioinformatics, 2014. **30**(10): p. 1363-9.

6. Calkins, M.E., et al., *The Philadelphia Neurodevelopmental Cohort: constructing a deep phenotyping collaborative.* J Child Psychol Psychiatry, 2015. **56**(12): p. 1356-1369.

7. Almstrup, K., et al., *Pubertal development in healthy children is mirrored by DNA methylation patterns in peripheral blood.* Sci Rep, 2016. **6**: p. 28657.

8. Hannon, E., et al., *An integrated genetic-epigenetic analysis of schizophrenia: evidence for co-localization of genetic associations and differential DNA methylation.* Genome Biol, 2016. **17**(1): p. 176.

9. Maksimovic, J., B. Phipson, and A. Oshlack, *A cross-package Bioconductor workflow for analysing methylation array data.* F1000Res, 2016. **5**: p. 1281.

10. Horvath, S., *DNA methylation age of human tissues and cell types.* Genome Biol, 2013. **14**(10): p. R115.

11. Chen, Y.A., et al., *Discovery of cross-reactive probes and polymorphic CpGs in the Illumina Infinium HumanMethylation450 microarray.* Epigenetics, 2013. **8**(2): p. 203-9.

12. Peters, T.J., et al., *De novo identification of differentially methylated regions in the human genome.* Epigenetics Chromatin, 2015. **8**: p. 6.

13. Reinius, L.E., et al., *Differential DNA methylation in purified human blood cells: implications for cell lineage and studies on disease susceptibility.* PLoS One, 2012. **7**(7): p. e41361.

14. Jaffe, A.E. and R.A. Irizarry, *Accounting for cellular heterogeneity is critical in epigenome-wide association studies.* Genome Biol, 2014. **15**(2): p. R31.

15. Auton, A., et al., *A global reference for human genetic variation.* Nature, 2015. **526**(7571): p. 68-74.
